# Synaptic network structure shapes cortically evoked spatio-temporal responses of STN and GPe neurons in a computational model

**DOI:** 10.3389/fninf.2023.1217786

**Published:** 2023-08-22

**Authors:** Justus A. Kromer, Hemant Bokil, Peter A. Tass

**Affiliations:** ^1^Department of Neurosurgery, Stanford University, Stanford, CA, United States; ^2^Boston Scientific Neuromodulation, Valencia, CA, United States

**Keywords:** basal ganglia, evoked responses, functional channels, network connectivity, multichannel stimulation, neural networks

## Abstract

**Introduction:**

The basal ganglia (BG) are involved in motor control and play an essential role in movement disorders such as hemiballismus, dystonia, and Parkinson's disease. Neurons in the motor part of the BG respond to passive movement or stimulation of different body parts and to stimulation of corresponding cortical regions. Experimental evidence suggests that the BG are organized somatotopically, i.e., specific areas of the body are associated with specific regions in the BG nuclei. Signals related to the same body part that propagate along different pathways converge onto the same BG neurons, leading to characteristic shapes of cortically evoked responses. This suggests the existence of functional channels that allow for the processing of different motor commands or information related to different body parts in parallel. Neurological disorders such as Parkinson's disease are associated with pathological activity in the BG and impaired synaptic connectivity, together with reorganization of somatotopic maps. One hypothesis is that motor symptoms are, at least partly, caused by an impairment of network structure perturbing the organization of functional channels.

**Methods:**

We developed a computational model of the STN-GPe circuit, a central part of the BG. By removing individual synaptic connections, we analyzed the contribution of signals propagating along different pathways to cortically evoked responses. We studied how evoked responses are affected by systematic changes in the network structure. To quantify the BG's organization in the form of functional channels, we suggested a two-site stimulation protocol.

**Results:**

Our model reproduced the cortically evoked responses of STN and GPe neurons and the contributions of different pathways suggested by experimental studies. Cortical stimulation evokes spatio-temporal response patterns that are linked to the underlying synaptic network structure. Our two-site stimulation protocol yielded an approximate functional channel width.

**Discussion/conclusion:**

The presented results provide insight into the organization of BG synaptic connectivity, which is important for the development of computational models. The synaptic network structure strongly affects the processing of cortical signals and may impact the generation of pathological rhythms. Our work may motivate further experiments to analyze the network structure of BG nuclei and their organization in functional channels.

## 1. Introduction

The BG are a sub-cortical complex that consists of several nuclei, such as the subthalamic nucleus (STN) and the globus pallidus (Soghomonian and Jagaroo, 2016). Due to different synaptic projections, the latter is divided into internal (GPi) and external segments (GPe). The BG play an important role in decision-making, motor control, and motor learning. Several neurological disorders are associated with abnormal BG activity, such as excessive synchronization in Parkinson's disease, and alternations in synaptic connectivity (Hammond et al., 2007; Madadi Asl et al., 2022b). The STN and GPe circuit is in the center of the BG and is believed to be critical for the generation of oscillations (Plenz and Kital, 1999; Bevan et al., 2002; Crompe et al., 2020). Furthermore, the STN is a major target for high-frequency deep brain stimulation, the current standard of care for medically refractory Parkinson's disease (Krack et al., 2003).

**Figure 1:**
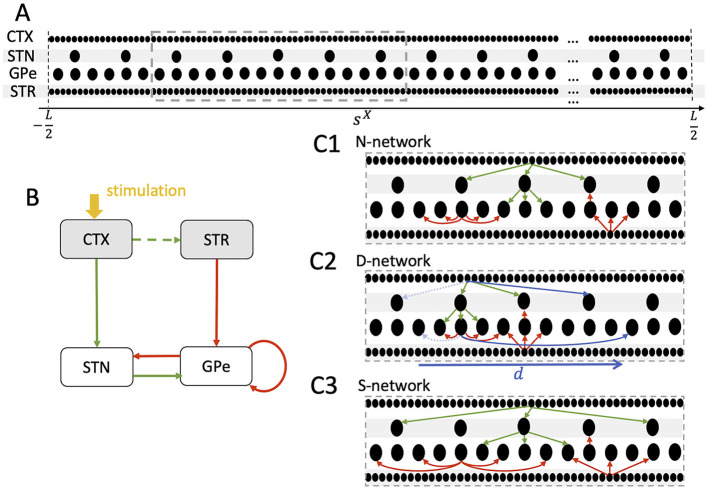
Schematics of neuron placement and the three network structures considered throughout the paper. **(A)** Neurons (black dots) were placed in the interval [−*L*/2, *L*/2] (Model and methods). Similar coordinates, *s*^X^, refer to neurons representing similar body parts. A total of 10^3^ cortical (CTX), 100 STN, 300 GPe, and 10^3^ striatal (STR) medium spiny neurons (MSN) was considered. **(B)** Schematic of the STN-GPe circuit and its CTX and STR Poisson input (gray). Green arrows indicate glutamatergic and red arrows GABAergic synaptic interaction. Stimulation (yellow) is delivered to the CTX Poisson spike generators and leads to a transient increase of spiking activity in the cortex. Excitatory CTX input to the striatum is modeled by increasing STR MSN activity following CTX stimulation (see Model and methods). **(C1–C3)** Schematics of *nearest postsynaptic neurons* networks (N-networks) **(C1)**, *displaced postsynaptic neurons* networks (D-networks) **(C2)**, and *skip postsynaptic neurons* networks (S-networks) **(C3)**. A small number of connections of each type are shown for a single presynaptic neuron in a small portion of the network (dashed gray box in **A**). The actual numbers of connections are given in **Table 2**. In N-networks **(C1)**, neurons project to postsynaptic neurons at similar coordinates. In D-networks **(C2)**, 10% of the synaptic connections are randomly selected to connect to postsynaptic neurons with coordinates shifted by *d* (blue, see Model and Methods). Connections before shifting are marked by dashed light blue arrows. In S-networks **(C3)**, neurons project to postsynaptic neurons with similar coordinates except that every second postsynaptic neuron is skipped.

The STN receives topographically organized glutamatergic inputs from the cerebral cortex via the cortico-STN hyperdirect pathway, and gamma-aminobutyric acid (GABA)ergic inputs from the GPe via the cortico-striato-GPe-STN indirect pathway (see Figure 1) (Jeon et al., 2022). Additionally, synaptic input to the BG nuclei is organized somatotopically (Nambu, 2011), i.e., motor cortical neurons in regions representing different body parts project to different regions in these nuclei (Monakow et al., 1978; Nambu et al., 1996, 2002; Miyachi et al., 2006). On the other hand, BG neurons respond to motor cortex stimulation (Nambu et al., 2000; Kita and Kita, 2011; Polyakova et al., 2020) and to active and passive movement of corresponding body parts (DeLong et al., 1985). These characteristics are harnessed during stereotaxic surgery for electrode placement for deep brain stimulation as a treatment for movement disorders such as Parkinson's disease (Kaplitt et al., 2003; Krack et al., 2003).

Experimental studies in primates and rodents analyzed the response of STN and GPe neurons to electrical stimulation of different cortical areas, including the limb regions of the motor cortex, the primary sensory cortex, and the supplementary motor area (Nambu et al., 2000; Kita et al., 2004; Kita and Kita, 2011; Polyakova et al., 2020). The effect of local injections of glutamate and GABA antagonists into the STN (Polyakova et al., 2020), the GPe (Kita et al., 2004) as well as into the putamen and the GPe (Polyakova et al., 2020) on evoked responses was studied to get further insight into the involved pathways (Kita et al., 2004; Jaeger and Kita, 2011; Polyakova et al., 2020). Responding STN neurons showed complex response patterns characterized by an early and a late excitation followed by a late inhibition. These patterns indicated that signals from the stimulated cortical region reach STN neurons via two pathways: the monosynaptic cortico-STN pathway and the polysynaptic cortico-striato-GPe-STN pathway (Nambu et al., 2000; Kita and Kita, 2011; Polyakova et al., 2020). Furthermore, responding GPe neurons show characteristic responses consisting of an early excitation, an inhibition, and a late excitation (Nambu et al., 2000; Jaeger and Kita, 2011; Kita and Kita, 2011). The analysis of these evoked responses revealed the complex interplay of synaptic pathways in the cortico-basal ganglia circuit.

Evidence from animal models suggests that Parkinson's disease is not only accompanied by abnormal neuronal synchrony (Hammond et al., 2007) but also by alterations of synaptic connectivity in the BG (Fan et al., 2012; Chu et al., 2015, 2017; Madadi Asl et al., 2022b) and impaired somatotopy (Filion et al., 1988; Boraud et al., 2000; Cho et al., 2002). Furthermore, characteristic features of cortically evoked responses change in 6-hydroxydopamine (6-OHDA) lesioned rats, an animal model for Parkinson's disease (Kita and Kita, 2011). Besides Parkinson's disease, abnormal alterations of the somatotopic organization of the BG and their cortical inputs have been observed in other movement disorders (Bronfeld and Bar-Gad, 2011), such as motor tics (McCairn et al., 2009), appearing as a symptom, for instance, in Tourette syndrome, and dystonia (Tamburin et al., 2002; Delmaire et al., 2005). This suggests that alterations of synaptic connectivity shape cortically evoked responses, and likely affect the processing of cortical stimuli.

In the present paper, we build on these results and explore to which extent cortically evoked responses of STN and GPe neurons can be used to infer characteristics of the underlying synaptic network structure. In a computational model, we show that a characteristic width of parallel “functional channels” in the BG, which allow for parallel processing of multiple stimulation-induced cortical inputs, can be obtained based on the cortically evoked responses of STN and GPe neurons. Considering the underlying channel structure may be advantageous for the parameter adjustment and stimulation contact usage during multisite deep brain stimulation, for instance, for the delivery of coordinated reset stimulation in animal models for Parkinson's disease (Tass et al., 2012; Wang et al., 2016, 2022; Bore et al., 2022) or Parkinson's disease patients (Adamchic et al., 2014).

To study the relation between synaptic connectivity and cortically evoked responses, we developed a computational model of the BG that incorporates a simplified type of somatotopy (Nambu, 2011), where neurons tend to project to neurons that represent similar body parts characterized by similar (spatial) coordinates, as well as two modified somatotopy variants. Our model produces cortically evoked responses that mimic experimental data from rats (Kita and Kita, 2011). We analyzed the spatio-temporal pattern of cortically evoked responses and explored how it is affected by perturbations of the synaptic network structure. To quantify the width of parallel functional channels in the BG, we suggest a two-site stimulation approach in which two cortical stimuli cause two evoked responses in the STN and GPe. We quantify the modulation of the evoked response to a test stimulus by the presence of a priming stimulus and show how an approximate channel width can be inferred. The latter measures the minimum distance between cortical areas whose input to the BG is processed independently.

The present paper is organized as follows. First, we introduce our computational model and present details on the incorporated experimental data on synaptic connectivity as well as the suggested two-site stimulation technique. Next, we show that our computational model reproduces the experimentally observed characteristic sequence of excitations and inhibitions. Then, we analyze the spatio-temporal response pattern and study how it is affected by variations of the synaptic network structure. We present simulation results on evoked responses of STN and GPe neurons during two-site cortical stimulation and show how an estimate of the width of functional channels in the cortico-BG network can be obtained. Finally, we discuss our results.

## 2. Model and methods

We developed a computational model of the STN-GPe circuit that accounts for topographically organized synaptic connections. Following earlier studies, individual neurons were modeled by adaptive quadratic integrated-and-fire neurons to ensure low computational costs (Lindahl et al., 2013; Fountas and Shanahan, 2017). The organization of synaptic connections was partly motivated by earlier computational studies (Terman et al., 2002; Hahn and McIntyre, 2010; Kumaravelu et al., 2016), partly based on experimental data on the synaptic connectivity in the STN-GPe circuit (Oorschot, 1996; Sadek et al., 2007; Baufreton et al., 2009; Kita and Kita, 2011; Koshimizu et al., 2013; Ketzef and Silberberg, 2021), and partly obtained from parameter optimization to reproduce experimentally observed mean firing rates of the neurons (Fountas and Shanahan, 2017). Note that firing rates of BG neurons in rats vary depending on the state, e.g., awake, anesthetized, resting. Whenever possible, we considered the data from Kita and Kita (2011) in anesthetized rats, as they provide a detailed analysis of cortically evoked responses.

### 2.1. Neural network model

#### 2.1.1. Dynamics of membrane potentials

Following the approach of Lindahl et al. (2013) and Fountas and Shanahan (2017), individual neurons were modeled using adaptive quadratic integrate-and-fire neurons. This class of models was found to reproduce a wide class of neuronal spiking and bursting behavior (Izhikevich, 2003). The dynamics of the membrane potential, *v*_*i*_, of the *i*th GPe neuron was modeled as follows (Fountas and Shanahan, 2017)


(1) $$\begin{array}{l} \begin{array}{lll} C_{i}^{\text{GPe}}\frac{d}{dt}v_{i} & = & k^{\text{GPe}}(v_{i}-v^{\text{r},\text{GPe}})(v_{i}-v^{\text{t},\text{GPe}})-u_{1i}+I_{\text{GPe}},\\ \;\frac{d}{dt}u_{1i} & = & a^{\text{GPe}}(b^{\text{GPe}}(v_{i}-v^{\text{r},\text{GPe}})-u_{1i}). \end{array} \end{array}$$

$C_{i}^{\text{GPe}}$ is the membrane capacitance, *I*_GPe_ the applied current, and *u*_1*i*_ is a slow recovery variable with time scale given by 1/*a*^GPe^. *v*^r, GPe^ is the resting potential and *v*^t, GPe^ corresponds to the threshold potential. The other parameters adjust the shape of the nullclines and were chosen according to Fountas and Shanahan (2017).

We considered the parameter set for GPe neurons that exhibited periods of high frequency discharges (referred to as “GPe type B” neurons in Fountas and Shanahan (2017). These correspond to prototypic GPe neurons which present the largest neuronal population in the GPe and project to the STN (Mallet et al., 2012; Abdi et al., 2015). Neurons with this type of dynamics were observed more often than others in experiments in monkeys at rest (≈85% of GPe neurons in DeLong, 1972). Abdi et al. found that about two-thirds of GPe neurons are prototypic neurons in dopamine-intact rats (Abdi et al., 2015).

Whenever the membrane potential passed a threshold $v_{\text{peak}}^{\text{GPe}}$, the state variables were reset, i.e., $v_{i}\to c^{\text{GPe}}$ and $u_{1i}\to u_{1i}+d^{\text{GPe}}$ (Fountas and Shanahan, 2017).

To describe the dynamics of the membrane potential of the *i*th STN neuron, an additional slow variable was introduced (Fountas and Shanahan, 2017):


(2) $$\begin{array}{l} C_{i}^{\text{STN}}\frac{d}{dt}v_{i}=k^{\text{STN}}(v_{i}-v^{\text{r},\text{STN}})(v_{i}-v^{\text{t},\text{STN}})-u_{1i}-w^{\text{STN}}u_{2i}\\ \;+\;I^{\text{STN}},\\ \;\frac{d}{dt}u_{1i}=a_{\text{STN}}(b^{\text{STN}}(v_{i}-v^{\text{r},\text{STN}})-u_{1i}),\\ \;\frac{d}{dt}u_{2i}={ã}^{\text{STN}}(G(v_{i}){\tilde{b}}^{\text{STN}}(v_{i}-{\tilde{v}}^{\text{r},\text{STN}})-u_{2i}). \end{array}$$

The first two equations describe the dynamics of the membrane potential and a recovery variable, similar to the dynamics of GPe neurons given in Equation (1). In addition, a second recovery variable *u*_2*i*_ is used to describe the dynamics of STN neurons. *G*(*v*_*i*_) is the Heaviside step function, $H\left({ṽ}^{\text{r},\text{STN}}-v_{i}\right)$, such that *u*_2*i*_ activates below ṽ^r, STN^ and causes a rebound response (Fountas and Shanahan, 2017).

In rat brain slices, the majority of STN neurons was found to produce rebound burst firing after removal of a hyperpolarizing current (17 out of 20 neurons in Bevan et al., 2000). We modeled such STN neurons using the parameter set for rebound bursting STN neurons from Fountas and Shanahan (2017).

Whenever the membrane potential passed a threshold $v_{\text{peak}}^{\text{STN}}+Uu_{2i}$, the state variables of STN neurons were reset $v_{i}\to c^{\text{STN}}-Uu_{2i}$, $u_{1i}\to u_{1i}+d^{\text{STN}}$, and $u_{2i}\to u_{2i}+{\tilde{d}}^{\text{STN}}$ (Fountas and Shanahan, 2017). Here, $U={\left(w^{\text{STN}}|u_{2i}|+1/w^{\text{STN}}\right)}^{-1}$ (Fountas and Shanahan, 2017).

To ensure heterogeneity, the membrane capacitances of neurons of each type X=STN or X=GPe were distributed according to a Gaussian distribution with mean $\langle C_{j}^{\text{X}}\rangle$ and standard deviation $0.1\langle C_{j}^{\text{X}}\rangle$. A complete list of the parameter values used to model GPe and STN neurons can be found in Table 1.

**Table 1:** Parameters for single-neuron dynamics according to Fountas and Shanahan (2017).

	**GPe**	**STN**
$\langle C_{j}^{\text{STN / GPe}}\rangle$ (pF)	68.0	23.0
*k*^STN / GPe^ (nS/mV)	0.943	0.439
*v*^*r*, STN / GPe^ (mV)	−53.0	−56.2
ṽ^*r*, STN / GPe^ (mV)		−60.0
*v*^*t*, STN / GPe^ (mV)	−44.0	−41.4
$I_{\text{bias}}^{\text{STN / GPe}}$ (pA)	64.0	56.1
*w* ^STN / GPe^		0.1
$\tilde{w^{\text{STN / GPe}}}$		0.0
*a*^STN / GPe^ (1/ms)	0.0045	0.021
*b*^STN / GPe^ (nS)	3.895	4.0
ã^STN / GPe^ (1/ms)		0.123
${\tilde{b}}^{\text{STN / GPe}}$ (nS)		0.015
$v_{\text{peak}}^{\text{STN / GPe}}$ (mV)	25.0	15.4
*c*^STN / GPe^ (ms)	−58.36	−47.7
*d*^STN / GPe^ (pA)	0.353	17.1
${\tilde{d}}^{\text{STN / GPe}}$ (pA)		−68.4
θ^STN / GPe^	3.0	0.5

Cortical (CTX) neurons and striatal medium spiny neurons (MSN)s expressing D2 receptors were modeled as Poisson spike generators with baseline firing rates *r*^CTX^ and *r*^MSN^, respectively. We used *r*^CTX^ = 4 Hz since Dejean et al. (2008) reported 4.1 ± 1.3 spikes per second in freely moving rats. In addition, we selected *r*^MSN^ = 0.67 Hz, representing the firing rate of spontaneously active medium spiny D2 neurons in anesthetized rats in the dopamine-intact state in Kita and Kita (2011). Note that *r*^MSN^ is well in the range of 0.8 ± 0.2 Hz reported by Dejean et al. (2008) for freely moving rats.

In our computational model, we simulated 10^3^ CTX and 10^3^ MSN Poisson spike generators that provided synaptic input to the STN and GPe, respectively. The STN consisted of 100 neurons and the GPe of 300 neurons. The ratio of the total numbers of STN and GPe model neurons was selected to reproduce the ratio observed in young adult rats by Oorschot (1996). There, the total number of STN neurons was estimated as (13.56 ± 1.41) × 10^3^ (mean ± std.) and the total number of GPe neurons as (45.96 ± 5.12) × 10^3^ (mean ± std.).

#### 2.1.2. Synaptic dynamics

To model synaptic connections, we considered the time-dependent conductances $g_{j}^{\text{X,Y}}\left(t\right)$, with dynamics given by


(3) $$\begin{array}{l} \tau^{\text{X,Y}}\frac{dg_{j}^{\text{X,Y}}}{dt}=-g_{j}^{\text{X,Y}}. \end{array}$$

τ^X, Y^ is the synaptic time scale. $g_{j}^{\text{X,Y}}\left(t\right)$ describes the total input conductance for synaptic inputs from neurons in nucleus X to the neuron *j* in nucleus Y. $g_{j}^{\text{X,Y}}$ was increased instantaneously at all spike arrival times: $g_{j}^{\text{X,Y}}\to g_{j}^{\text{X,Y}}+G^{\text{X,Y}}$, at times $t_{i}+\lambda^{\text{X,Y}}$. Here, *t*_*i*_ is the spike time of a presynaptic neuron and λ^X, Y^ is the synaptic transmission delay between presynaptic spike time and the arrival of the action potential at the synapse.

We considered GABAergic synapses and two types of receptors for glutamatergic synapses: alpha-amino-3-hydroxy-5-methyl-4-isoxazolepropionic acid (AMPA) receptors and slower N-methyl-D-aspartic acid (NMDA) receptors. As GABA and AMPA are considered rather fast, we neglect the rise time of the corresponding synaptic conductances. The resulting postsynaptic currents were given by Fountas and Shanahan (2017)


(4) $$\begin{array}{l} I_{\text{Z},j}^{\text{X,Y}}=g_{j}^{\text{X,Y}}\left(E^{\text{X,Y}}-v_{j}\right),\;\text{Z}=\text{GABA,AMPA}. \end{array}$$

*E*^X, Y^ is the synaptic reversal potential. The dynamics of $g_{j}^{\text{X,Y}}$ is given by Equation (3) and the corresponding τ^X, Y^ is the decay time of the synaptic potentials.

In contrast, NMDA receptors are rather slow and the rise time of the corresponding conductance is of the order of the decay times for GABA and AMPA currents (Kumaravelu et al., 2016). We therefore modeled the rise and the decay of the corresponding synaptic conductances after each spike arrival:


(5) $$\begin{array}{l} I_{Z,j}^{\text{X,Y}}\left(v_{j}\right)=\left(g_{\text{slow},j}^{\text{X,Y}}-g_{\text{fast},j}^{\text{X,Y}}\right)\left(E^{\text{X,Y}}-v_{j}\right),\;\text{Z}=\text{NMDA}. \end{array}$$

The dynamics of both the slow, $g_{\text{slow},j}^{\text{X,Y}}$, and fast conductance, $g_{\text{fast},j}^{\text{X,Y}}$, were given by Equation (3), and the corresponding synaptic time scales quantify the fast rise and the slow decay of the total synaptic conductance $g_{\text{slow},j}^{\text{X,Y}}-g_{\text{fast},j}^{\text{X,Y}}$, respectively, for the resulting postsynaptic currents. In addition, we considered a voltage-dependent magnesium plug for the NMDA receptors given by Fountas and Shanahan (2017)


(6) $$\begin{array}{l} B\left(v\right)=1.0/\left(1.0+0.28\exp\left(-0.062v\right)\right). \end{array}$$

The total postsynaptic current $I_{j}^{\text{Y}}$ into neuron *j* in Equations (1) and (2) was then given by


(7) $$\begin{array}{l} I_{j}^{\text{GPe}}=I_{\text{AMPA},j}^{\text{STN},\text{GPe}}(v_{j})+B(v_{j})\;I_{\text{NMDA},j}^{\text{STN},\text{GPe}}(v_{j})+I_{\text{GABA},j}^{\text{MSN},\text{GPe}}(v_{j})\\ \;+\;I_{\text{GABA},j}^{\text{GPe},\text{GPe}}(v_{j})+I_{\text{bias}}^{\text{GPe}}+\sqrt{2\theta^{\text{GPe}}}C_{j}^{\text{GPe}}\xi_{j}(t) \end{array}$$

and


(8) $$\begin{array}{l} \begin{array}{l} I_{j}^{\text{STN}}=I_{\text{AMPA},j}^{\text{CTX},\text{STN}}(v_{j})+B(v_{j})\;I_{\text{NMDA},j}^{\text{CTX},\text{STN}}(v_{j})+I_{\text{GABA},j}^{\text{GPe},\text{STN}}(v_{j})+I_{\text{bias}}^{\text{STN}}\\ \;+\;\sqrt{2\theta^{\text{STN}}}C_{j}^{\text{STN}}\xi_{j}(t), \end{array} \end{array}$$

respectively. Here, $I_{\text{bias}}^{\text{STN}}$ and $I_{\text{bias}}^{\text{GPe}}$ are constant bias currents that adjust the baseline activity of STN and GPe neurons, respectively. ξ_*j*_(*t*) is zero mean, white Gaussian noise with amplitude scaled by θ^X^. All parameter values related to the synaptic dynamics are given in Table 2.

**Table 2:** Parameters used to model synaptic interaction.

	**τ^X, Y^ (ms)**	**λ^X, Y^ (ms)**	***G*^X, Y^ (nS)**	***E*^X, Y^ (mV)**	***N* ^X, Y^**
CTX → STN (AMPA)	2 [1]	1.0^1^	opt.	0 [1]	3 [1]
CTX → STN (NMDA)	2 [3], 100 [1]	$\lambda_{\text{AMPA}}^{\text{CTX,STN}}$	$0.6\;G_{\text{AMPA}}^{\text{CTX,STN}}$	0 [1]	3 [1]
STN → GPe (AMPA)	2 [1]	1.0^2^	opt.	0 [1]	
STN → GPe (NMDA)	2 [3], 100 [1]	$\lambda_{\text{AMPA}}^{\text{STN,GPe}}$	$0.36\;G_{\text{AMPA}}^{\text{STN,GPe}}$	0 [1]	
GPe → GPe	5 [4]	5.0 [5]	opt.	−85 [3]	20 [6]
MSN → GPe	5 [3]	7.4 [5]^3^	opt.	−85[3]	10 [1]
GPe → STN	8 [4]	λ^STN, GPe^	opt.	−84 [4]	1 [7]
CTX → MSN		10.5 [2]			

For NMDA receptors, the first synaptic time scale τ^X, Y^ represents the rise and the second the decay time. Values obtained from numerical optimization (“opt.”) are given in Table 3. *N*^STN, GPe^ was a free parameter. References: [1] Fountas and Shanahan (2017), [2] Kita and Kita (2011), [3] Kumaravelu et al. (2016), [4] Lindahl et al. (2013). [5] Ketzef and Silberberg (2021), [6] Kita (2007), and [7] Baufreton et al. (2009).

1. Was adjusted such that the onset of the early excitation in cortically evoked response occurs at ~5 − 6 ms [2].

2. Was adjusted such that the onset of the early excitation in cortically evoked response occurs at ~7 − 10 ms [2].

3. Onset delay of prototypic neurons to striatial stimulation was 7.34 ± 0.35 ms and that of arkypallidal cells 8.6 ± 0.43 ms [5]. Here, we only considered prototypic neurons.

#### 2.1.3. Synaptic network structure

Synaptic connections in the BG are somatotopically organized (Nambu, 2011). To incorporate somatotopy in our computational model, we introduced coordinates *s*^X^, where X denotes the corresponding nucleus, as before. The maximal range of these coordinates is denoted by *L* and will be set to one. For a given neuron, *s*^X^ represents the *feature* that is represented, e.g., the body part or motor program. Similar coordinate values of different neurons refer to similar features. Alternatively, given that synaptic connections in the BG are organized somatotopically (Nambu, 2011), *s*^X^ can be interpreted as a spatial coordinate. Neurons in each nucleus were equidistantly placed in the interval [−*L*/2, *L*/2].

In our reference scenario, which will be referred to as N-network throughout the paper, neurons connect to postsynaptic neurons with similar features, i.e., *s*^X^≈*s*^Y^, where X refers to the presynaptic nucleus and Y to the postsynaptic nucleus. Thus, the somatotopic organization of synaptic connections is intact. In the computational model, we fixed the number of outgoing connections per neuron, *N*^X, Y^, according to the values given in Table 2. Below, we give more details on the choices of *N*^X, Y^. Then, for each presynaptic neuron, we chose the postsynaptic neurons such that the difference in coordinates |*s*^Y^−*s*^X^| was minimal among all possible postsynaptic neurons.

Parkinson's disease and other neurological disorders impact many aspects of the nervous system. Here, as discussed below, we focus on synaptic reorganization. We compared the results for N-networks with perturbed network structures in which a portion of synaptic connections was rearranged. Specifically, we considered D-networks and S-networks, which were constructed as follows.

- **D-networks:** The first type of perturbation of N-networks was a *displacement* of a fraction of connections. Specifically, we randomly selected a portion P of the connections and rearranged them such that |(*s*^Y^−*d*)−*s*^X^| was minimal (see Figure 1C2 for an illustration). Thus, these connections then targeted postsynaptic neurons that were displaced by *d*. Throughout the present paper, we chose P=0.1 and *d* = 0.15*L*. Connections were randomly selected for displacement according to a uniform probability distribution. D-networks mimic the situation were the somatotopy is perturbed such that a region representing a certain body part also forms projections to a region that represents a different body part. This was motivated by results in the 1-methyl-4-phenyl-l,2,3,6-tetrahydropyridine (MPTP) monkey model of Parkinson's disease, where pallidal neurons in control conditions responded to movement of a single joint but neurons responded to movement of multiple joints after MPTP intoxication (Filion et al., 1988; Boraud et al., 2000; Pessiglione et al., 2005; Bronfeld and Bar-Gad, 2011).
- **S-networks:** The second type of perturbation of the network structure was a skipping of neurons in the postsynaptic nucleus (see Figure 1C3 for an illustration). This led to an increase in the projection area in the postsynaptic nucleus. In the present paper, we considered the case where every second postsynaptic neuron was skipped. Thus, synaptic connections were implemented as in N-networks, except that each presynaptic neuron projected only to every second postsynaptic neuron, starting with the one for which |*s*^Y^−*s*^X^| was minimal (see Figure 1C3). This network structure was motivated by experimental studies on striatal neurons in the 6-OHDA rat model for Parkinson's disease (Cho et al., 2002). There, neurons related to a certain body part occurred in clusters in healthy controls. After 6-OHDA lesion, the cluster size shrank and some of the neurons at the borders became related to different body parts, suggesting a larger overlap of regions representing different body parts.

To ensure that the obtained network did not depend on the order in which synaptic connections were added between neurons, we added small random offsets to the neurons' coordinates that were uniformly distributed between zero and 10^−4^*L* for CTX and MSN Poisson spike generators and between zero and 10^−3^*L* for STN and GPe neurons. This way, there were no two neuron pairs that had identical distances to each other. Then, pairs of pre- and postsynaptic neurons were sorted according to the distances between them, and synaptic connections were added.

The numbers of outgoing connections per presynaptic neuron, *N*^X, Y^, were either motivated by experimental data, taken from earlier computational studies, or obtained from parameter optimization.

- For the outgoing synaptic connections of the two populations of Poisson spike generators, we chose *N*^CTX, STN^ = 3 and *N*^MSN, GPe^ = 10, which reproduced the connection probabilities used for random connections in Fountas and Shanahan (2017), where the connection probability for CTX to STN connections was 0.03 and the connection probability for MSN to GPe connections was 0.033.
- Baufreton et al. (2009) studied GPe → STN connections. They found that these connections were sparse but highly selective. Based on the number of synaptic boutons per GPe neuron in the STN, they estimated that each GPe neuron forms only enough synaptic boutons to contact <2% of the STN neurons. Furthermore, they reported that GPe neurons form many synapses with each postsynaptic STN neuron. Their data also suggest that neighboring STN neurons rarely receive input from the same GPe neuron. Based on these findings, we chose *N*^GPe, STN^ = 1.
- Kita (2007) observed that large areas of somata and dendrites of the GPe projection neurons are covered with synaptic boutons. The majority of which belonged to striatal axons. We chose *N*^GPe, GPe^ = 20 outgoing GPe → GPe connections per GPe neuron such that the majority of GABAergic synapses came from striatal neurons.
- The numbers of outgoing STN → GPe connections differ substantially among previous computational models. Hahn and McIntyre (2010) considered rather focused projections of STN neurons to GPe neurons, resembling a high degree of specificity of STN → GPe connections in functionally related areas in the GPe as observed experimentally in monkeys (Shink et al., 1996). In their computational model, STN neurons only project to GPe neurons in the same channel, i.e., each STN neuron projected to the three closest GPe neurons (*N*^STN, GPe^ = 3). Other computational studies considered more diffuse STN → GPe connections, e.g., in Fountas and Shanahan (2017) each STN neuron projected to 30% of the GPe neurons (corresponding to 90 STN → GPe connections per STN neuron). In Lindahl et al. (2013), each STN neuron had 30 STN → GPe connections. These latter numbers were motivated by experimental data on the organization of STN and STR synaptic terminals in the GPe obtained from earlier labeling studies in monkeys (Hazrati and Parent, 1992; Parent and Hazrati, 1993). In these studies, it was suggested that STN → GPe and STR → GPe connections are highly organized and that STN excitation targets larger groups of GPe neurons. In contrast, STR inhibition specifically targets subsets of these groups. Later, STN projections were studied in more detail in rat brain segments (Koshimizu et al., 2013). There, STN neurons were found to form large numbers of axon boutons inside the GPe. Furthermore, boutons were highly clustered in groups indicating projections to localized groups of pallidal neurons. Furthermore, there was high variability in the number of axon boutons formed per STN neuron. Throughout the present paper, we varied the number of STN-GPe connections to study to which extent our results depend on *N*^STN, GPe^. Specifically, we considered the cases *N*^STN, GPe^ = 3 (Hahn and McIntyre, 2010) and *N*^STN, GPe^ = 30 (Lindahl et al., 2013), spanning the range from highly focused (each STN neurons targets 1% of GPe neurons) onto a small cluster of GPe neurons to diffuse projections onto a macroscopic portion of the GPe (each STN neurons targets 10% of GPe neurons).

The values of all maximal synaptic conductances, *G*^X, Y^, were chosen such that experimental data for the stationary mean firing rate of STN and GPe neurons were reproduced. In particular, we followed Fountas and Shanahan (2017) and considered a sequence of scenarios in which different neuronal populations were inhibited. Following, we describe the resulting parameter adjustment algorithm:

1. **Estimation of** ***G***^**CTX, STN**^**:** In the first step, we applied a parameter optimization algorithm to find values of *G*^CTX, STN^ such that the mean firing rate of STN neurons was close to experimental data from Farries et al. (2010) in rats *in vivo*. In these rats, a large excitotoxic lesion was applied to the GPe. In our computational model, this was implemented by considering only the CTX spike generators and the STN neurons, i.e., the STN was isolated from the GPe by setting *G*^GPe, STN^ = 0 nS. Farries et al. (2010) reported STN firing rates of 20.7 ± 5.2 Hz during these experiments, which was about twice as large as the firing rate of STN neurons measured in rats with intact GPe (9.5 ± 3.5 Hz). The parameter optimization procedure was performed as follows: The firing rate of STN neurons was estimated by performing simulations of the computational model for 40 s and 12 different initial conditions. Each initial condition had a random realization of membrane capacitances, initial values of membrane potentials, and slow variables. To reduce finite size effects, neurons close to the borders of the interval for *s*^X^ were ignored and only spikes of the center third of STN neurons were recorded (STN neurons with indices 33 − 66) during the time interval *t*∈[30, 40) s. Then, an estimate of the average mean firing rate $r_{\text{est}}^{\text{STN}}$ was obtained based on the spike count. These simulations were repeated for different values of *G*^CTX, STN^ and the difference between $r_{\text{est}}^{\text{STN}}$ and 20.7 Hz (Farries et al., 2010) was minimized using the python function “scipy.optimize.minimize” (scipy version 1.5.4) with “Nelder-Mead” method and a tolerance of 0.1. We used *G*^CTX, STN^ = 1 nS as the initial guess. This procedure led to *G*^CTX, STN^ = 0.125 nS.
2. **Estimation of** ***G***^**GPe, STN**^**:** To estimate the value of *G*^GPe, STN^, we followed the approach of Fountas and Shanahan (2017) and added the GPe to the model from (1) using *G*^CTX, STN^ = 0.125 nS. For this step, the GPe neurons were modeled by a population of 300 Poisson spike generators with mean firing rate of 30.4 Hz (Kita and Kita, 2011, anesthetized rats). Then, we applied a similar algorithm as described in the previous paragraph optimizing the value of *G*^GPe, STN^ such that the firing rate of STN neurons became close to 11.8 Hz. Kita and Kita (2011) measured 11.8 ± 9.1 Hz in rats that were anesthetized with isoflurane. The initial guess was *G*^GPe, STN^ = 6.82 nS which was the peak conductance measured by Baufreton et al. (2009). Baufreton et al. reported a range for *G*^GPe, STN^ of 0.51 − 25.33 nS). This optimization led to *G*^GPe, STN^ = 1.11 nS
3. **Estimation of** ***G***^**STN, GPe**^**:** Next, we adjusted the parameters *G*^STN, GPe^ and *N*^STN, GPe^. For *N*^*STN, GPe*^, we considered two values that were taken from previous computational studies: *N*^STN, GPe^ = 3 (Hahn and McIntyre, 2010) and *N*^STN, GPe^ = 30 (Lindahl et al., 2013). For both values of *N*^STN, GPe^, *G*^STN, GPe^ was adjusted by considering the CTX-STN-GPe network without GABAergic inputs to GPe neurons. Celada et al. (1999) found that local bicuculline infusion, a GABA antagonist, into the globus pallidus of anesthetized rats led to a ≈55% increase of the mean firing rate of neurons in the globus pallidus. Note that the firing rate of these neurons in anesthetized rats might differ from the one in awake rats. Motivated by these experiments, we performed a similar optimization algorithm as in the previous paragraphs. During optimization, we replaced the STN neurons with a population of Poisson neurons firing with a mean firing rate of 11.8 Hz. This implicitly assumed that altered spiking of pallidal neurons in response to local bicuculline infusion had little effect on the majority of STN neurons. During optimization, the value of *G*^STN, GPe^ was varied such that GPe firing rates were 55% higher than in control conditions. For the control case, we used the firing rates from Kita and Kita (2011), who measured 30.4 ± 11.4 Hz in rats anesthetized with isoflurane. Thus, our target firing rate for GPe neurons was 47.12 Hz. To tune *G*^STN, GPe^, we ran the parameter optimization algorithm to find a value of *G*^STN, GPe^ for which the GPe firing rate was close to the target values. For *N*^STN, GPe^ = 3 the algorithm led to *G*^STN, GPe^ = 15.8 nS for which we obtained $r_{\text{est}}^{\text{GPe}}\approx 47.1$ Hz. For *N*^STN, GPe^ = 30, we found *G*^STN, GPe^ = 1.5 nS resulting in $r_{\text{est}}^{\text{GPe}}\approx 47.0$ Hz.
4. **Estimation of** ***G***^**MSN, GPe**^ **and** ***G***^**GPe, GPe**^**:** For the two different values of *N*^STN, GPe^ described in the previous paragraph and their corresponding values of *G*^STN, GPe^, we searched for values of the maximal conductances *G*^MSN, GPe^ and *G*^GPe, GPe^ such that the STN firing rate was close to the target value 11.8 Hz (Kita and Kita, 2011, anesthetized rats) and the GPe firing rate was close to the target value 30.4 Hz (Kita and Kita, 2011, anesthetized rats) in the intact STN-GPe circuit (Figure 1). We minimized

(9) $$\begin{array}{l} \text{Δ}R=|\frac{r^{\text{STN}}-11.8\text{Hz}}{\sigma_{\text{STN}}}|+|\frac{r^{\text{GPe}}-30.4\text{Hz}}{\sigma_{\text{GPe}}}|, \end{array}$$

with σ_STN_ = 9.1 Hz and σ_GPe_ = 11.4 Hz being the estimated standard deviations of single neuron baseline firing rates obtained from Kita and Kita (2011) (anesthetized rats) (see Tables 1, 2 in Kita and Kita, 2011). Using a similar optimization algorithm as in the previous paragraphs, we found that *G*^MSN, GPe^ = 5.54 nS and *G*^GPe, GPe^ = 0.44 nS minimized Δ*R* for *N*^STN, GPe^ = 3 (the resulting firing rates were $r_{\text{est}}^{\text{STN}}\approx 13.6$ Hz and $r_{\text{est}}^{\text{GPe}}\approx 30.5$ Hz). For *N*^STN, GPe^ = 30, we found that *G*^MSN, GPe^ = 12.0 nS and *G*^GPe, GPe^ = 0.21 nS minimized Δ*R*, which led to $r_{\text{est}}^{\text{STN}}\approx 11.8$ Hz and $r_{\text{est}}^{\text{GPe}}\approx 30.4$ Hz.

### 2.2. Cortical stimulation

Cortical stimulation was modeled by temporally increasing the firing rate of cortical Poisson spike generators. We implemented a spatial stimulus profile that determined the probability $P\left(s_{i}^{\text{CTX}}|s_{0}\right)$ at which a cortical Poisson spike generator at coordinate $s_{i}^{\text{CTX}}$ spikes in response to a stimulus delivered to *s*_0_


(10) $$\begin{array}{l} P\left(s_{i}^{\text{CTX}}|s_{0}\right)={\left(1+{\left(\frac{s_{i}^{\text{CTX}}-s_{0}}{\sigma_{\text{s}}}\right)}^{2}\right)}^{-1}. \end{array}$$

This profile was motivated by the shape of the profile of electrical stimuli used in Lysyansky et al. (2013). σ_s_ is the width of the stimulus profile and will be set to σ_s_ = 0.05*L*/π if not mentioned otherwise.

In experiments, cortical stimulation of the limb region resulted in a response of STR MSNs (Kita and Kita, 2011). We modeled the effect of the cortex on MSN activity by modifying the firing rate of the MSNs in response to afferent cortical neurons. Specifically, the MSN spike generator that was the closest to a cortical spike generator that spiked at time *t*_0_ in response to the stimulus, fired a spike between time *t* and time *t*+*h* with probability


(11) $$\begin{array}{l} P(t|s_{i}^{\text{MSN}},t_{0})=\\ \{\begin{array}{l} p(t-t_{0}-{\text{λ}}^{\text{CTX,MSN}})h,\;{\text{spike of closest cortical spike generator at t}}_{\text{0}}\\ r^{\text{MSN}},\text{ otherwise} \end{array} \end{array}$$

Here, *p*(*t*−*t*_0_) was chosen such that it approximated the shape of the probability density for a striatal spike after a stimulus at time 0 given in Figure 4A of Kita and Kita, 2011


(12) $$\begin{array}{l} p(t)=\{\begin{array}{l} \eta e^{\frac{-{\left(t-\mu \right)}^{2}}{2\sigma^{2}}},\;t<2\mu \\ 0,\;2\mu \leq t<2\mu +t_{\text{start}}\\ \frac{t-t_{\text{start}}}{t_{\text{end}}-t_{\text{start}}},\;2\mu +t_{\text{start}}\leq t<2\mu +t_{\text{end}}\\ \;r^{\text{MSN}},\;t\geq 2\mu +t_{\text{end}} \end{array},\;t>0, \end{array}$$

with η = 0.145, μ = 2.1, σ = μ/3. We used *t*_start_ = 100 ms and *t*_end_ = 300 ms.

**Figure 2:**
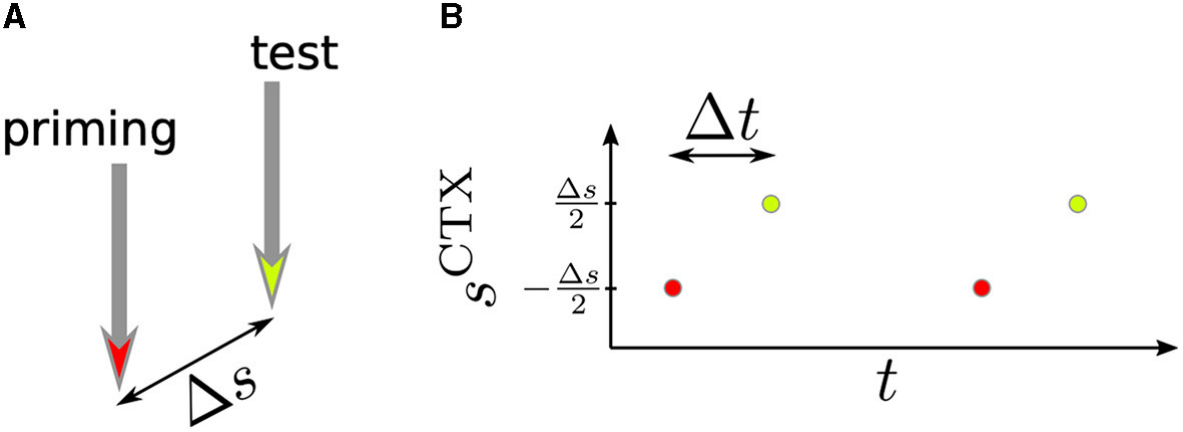
Schematics of two-pulse stimulation. **(A)** The priming stimulus (red) and the test stimulus (yellow) were delivered to two cortical locations. The distance between these locations was Δ*s*. **(B)** We delivered periodic sequences of priming and test stimuli and studied how the time lag, Δ*t*, and the distance between stimuli, Δ*s*, affected the response of BG neurons.

Cortical activation consisted of periodic sequences of 500 stimulus pulses delivered every 1.7 s (Kita and Kita, 2011). We also considered two-pulse stimulation where two pulses were delivered every 1.7 s. We refer to the first of the two pulses as *priming stimulus* and to the second pulse as *test stimulus*. The priming stimulus was centered at *s*^CTX^ = −Δ*s*/2 and the test stimulus was delivered after Δ*t* and centered at *s*^CTX^ = Δ*s*/2. Δ*s* and Δ*t* were varied. The two-pulse stimulation setup is illustrated in Figure 2.

### 2.3. Numerical details

Numerical integration was performed using the Euler–Maruyama method (Kloeden and Platen, 1992) with an integration time step of 0.05 ms. Numerical integration and data analysis was done in python. The times when the dynamics of individual neurons was reset were considered as the spike times.

**Figure 3:**
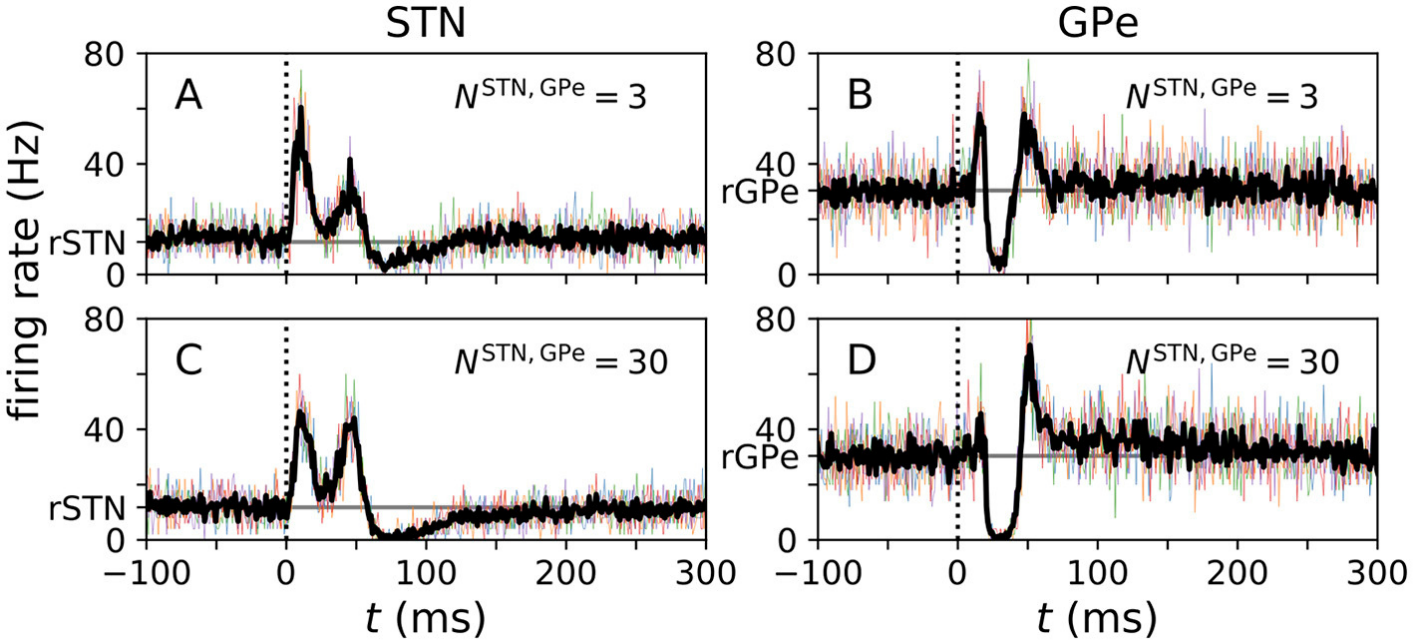
PSTHs obtained from computational model. **(A–D)** PSTHs for the center STN **(A, C)** and the center GPe **(B, D)** neuron obtained from simulations for the N-network. Colored curves show single-neuron PSTHs for five different trials. In each trial 500 stimuli were delivered and the center neurons' PSTHs were recorded. The black curves show averages of these trials. Simulations were performed for two N-networks with different numbers of STN to GPe connections, *N*^STN, GPe^, corresponding to a small projection area (*N*^STN, GPe^ = 3 as used in Hahn and McIntyre, 2010) and to a large projection area (*N*^STN, GPe^ = 30 as used in Lindahl et al., 2013), respectively. The vertical dotted line marks the stimulus delivery at *t* = 0 and the horizontal gray line marks the baseline firing rates *r*^STN^ and *r*^GPe^ in the absence of stimulation.

**Figure 4:**
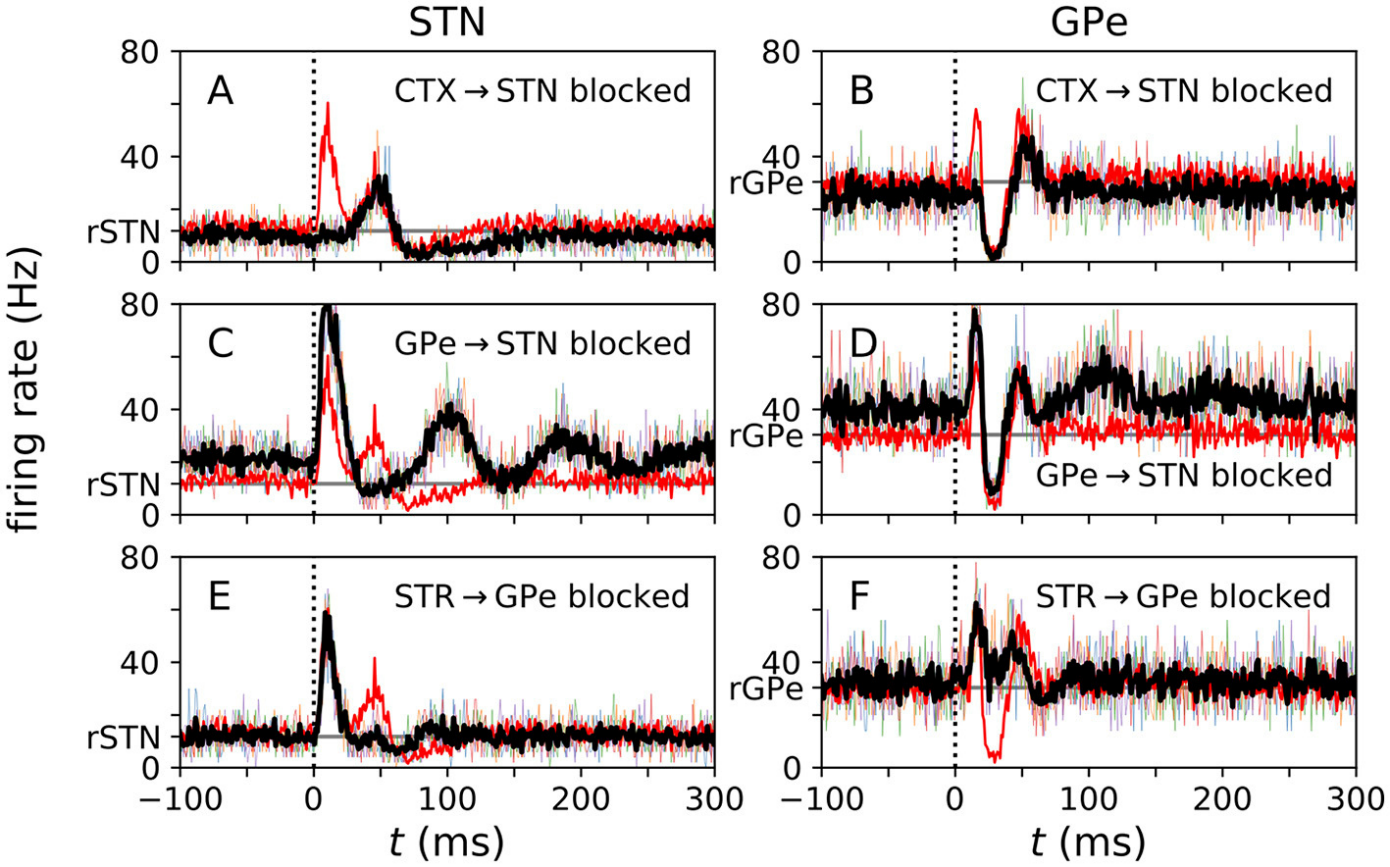
Local blockage of incoming connections affects evoked responses. **(A–F)** PSTHs for center STN **(A, C, E)** and center GPe **(B, D, F)** neurons obtained from simulations of the N-network, when incoming connections of respective types were blocked. Thin colored curves show single-neuron PSTHs from five different trials, each averaged over 500 stimuli. Black curve shows average over PSTHs from different trials. The red curve shows the control case (same as black curves in Figures 3A, B). Simulations were performed for three different cases: (top) all incoming cortical connections to the center three STN neurons were blocked; (center) all incoming GPe connections to the center three STN neurons were blocked; and (bottom) all incoming STR MSN connections to the center three GPe neurons were blocked. The vertical dotted line marks the stimulus delivery at *t* = 0 and the horizontal gray line marks baseline firing rates in the absence of stimulation. Parameters: *N*^STN, GPe^ = 3.

The peristimulus time histograms (PSTHs) in Figures 3, 4 were calculated as follows: first, simulations were run for five different trials, i.e., while the same realizations of single neuron parameters and network realizations were used in each trial, different realizations of the noisy input currents and Poisson inputs were considered. After, 40 s of simulated time the stimulation was started. A total of 500 stimuli was delivered for one-site stimulation. From the recorded spike trains, PSTHs were calculated by estimating the probability of a spike of the neuron in the very center of the *s*^X^ axes during a certain time bin of width 1 ms relative to the closest stimulus onset. Results are shown in Figures 3, 4.

To estimate the distributions of single-neuron mean firing rates (**Figure 6**), we performed simulations of 96 trials for each of the networks and each value of *N*^STN, GPe^. For each neuron, the mean firing rate was estimated by calculating the number of spikes during a time interval of nine seconds starting after 31 seconds of simulated time. Results in **Figure 6**, show a histogram of the single-neuron mean firing rates of the center 30 STN ($-1/6<s_{i}^{\text{STN}}<1/6$) and the center 100 GPe neurons ($-1/6<s_{i}^{\text{GPe}}<1/6$).

**Figure 5:**
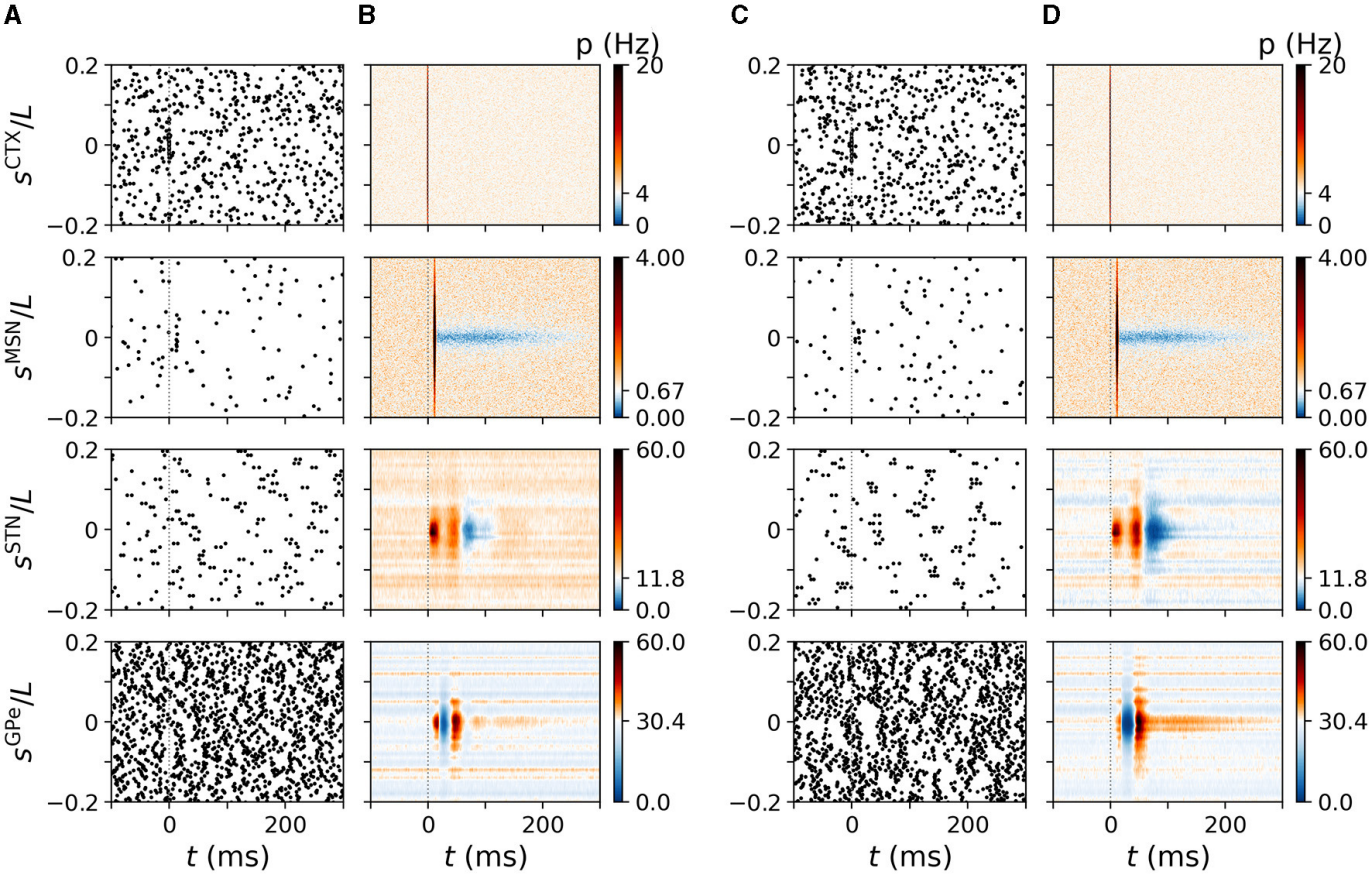
Cortically evoked spatio-temporal responses in a simulated N-network. Rows show responses of inner CTX (top), MSN (second to top), STN (second to bottom), and GPe neurons (bottom). Columns **(A, B)** show results of the computational model for *N*^STN, GPe^ = 3 and columns **(C, D)** show results for *N*^STN, GPe^ = 30. **(A)** Raster plots of spiking activity in the computational model triggered by a cortical stimulus at *t* = 0. *y*-axes shows neuron coordinates, *s*^X^. **(B)** trial-averaged instantaneous firing rate *p*(*t, s*^X^) of neurons at location *s*^X^ obtained by averaging over 500 stimuli and 24 realizations of noise and Poisson input. Color code indicates firing rate changes relative to baseline firing rate in the absence of stimulation (white). Increases in firing rate are shown in red/black and decreases in blue. **(C, D)** Same as **(A, B)** but for *N*^STN, GPe^ = 30. Parameters: *N*^STN, GPe^ = 3 **(A, B)** and *N*^STN, GPe^ = 30 **(C, D)**. CTX stimuli were centered at *s*^CTX^ = 0.

To estimate the spatio-temporal responses in Figures 5, **7, 8**, we performed simulations for 24 trials and calculated PSTHs as in Figures 3, 4 for neurons at different coordinates *s*^X^. From these PSTHs, the probability for a neuron with coordinate between *s*^X^ and *s*^X^+0.01/*L* (STN and GPe) and *s*^X^ and *s*^X^+0.001/*L* (CTX and MSN) at a time lag between *t* and *t*+1 ms relative to the closest stimulus onset was estimated by calculating the average number of spikes in that time and coordinate bin per trial and stimulus from the set of obtained single-neuron PSTHs. For one-site stimulation (Figures 5, **7**) 500 stimuli were delivered, as in Figures 3, 4. For two-site stimulation (**Figure 8**), 500 pairs of stimuli were delivered.

For each data point in **Figure 9**, we performed simulations similar to the ones in **Figure 8** for two scenarios. In the first scenario, one-site stimulation was delivered to the cortical location Δ*s*/2 and in the second one two-site stimulation was delivered to ±Δ*s*/2, respectively. Then, $L_{\text{base}}^{\text{X}}$ (Equation 13) and $L_{\text{re}}^{\text{X}}$ (Equation 14) were calculated as described in Section 3.6.

## 3. Results

Responses of STN and GPe neurons evoked by cortical stimulation were studied in monkeys (Nambu et al., 2000; Kita et al., 2004; Polyakova et al., 2020) and in rodents (Kita and Kita, 2011). Electrical stimuli were delivered to the motor cortex (Nambu et al., 2000; Kita et al., 2004; Kita and Kita, 2011; Polyakova et al., 2020) and the primary sensory cortex (Nambu et al., 2000) and PSTHs of responding STN and GPe neurons were recorded.

Responses of STN neurons showed an early and a late excitation followed by a long inhibition, whereas responses of GPe neurons showed an early excitation, an inhibition, and a late excitation. These characteristics were observed in rodents and in monkeys. Combining cortical stimulation with local drug injection, experiments in monkeys revealed that these characteristic features result from the interplay of two pathways: the cortico-STN glutamatergic hyperdirect pathway and the cortico-striato-GPe-STN indirect pathway (Kita et al., 2004; Kita, 2007; Jaeger and Kita, 2011; Polyakova et al., 2020).

Using our computational model, we explored how the characteristics of motor cortical stimulation-evoked responses depend on synaptic network connectivity. To this end, we mimicked the experimental setup in our computational model and studied PSTHs of STN and GPe neurons. We delivered cortical stimulation (Model and methods). Cortical stimuli were centered at *s*^CTX^ = 0, if not mentioned otherwise.

### 3.1. Evoked responses in computational model

PSTHs obtained from simulations of our computational model are shown in Figure 3. PSTHs of STN and GPe neurons show the typical characteristics observed in experiments. In particular, the characteristic sequence of an early excitation, a late excitation, and a long inhibition in responding STN neurons (Figures 3A, C) and the sequence of an early excitation, an inhibition, and a late excitation in responding GPe neurons (Figures 3B, D) were reproduced by our computational model.

The number of STN → GPe connections had a strong impact on how well the individual features were pronounced. The early excitation in GPe neurons was most pronounced for *N*^STN, GPe^ = 3 (Figure 3B), whereas it became less pronounced for large *N*^STN, GPe^ (Figure 3D). This was because the model with *N*^STN, GPe^ = 3 had stronger excitatory STN to GPe connections. The corresponding maximal conductance was chosen such that the STN and GPe firing rates were close to experimental data. Consequently, a small number of STN inputs strongly excited postsynaptic GPe neurons (see Table 3).

**Table 3:** Parameters for network connectivity.

**Parameter**	**Value**	**Source**
*N* ^CTX, STN^	3	(Fountas and Shanahan, 2017)
*N* ^MSN, GPe^	10	(Fountas and Shanahan, 2017)
*N* ^GPe, STN^	1	(Baufreton et al., 2009)
*N* ^GPe, GPe^	20	estimated, (Sadek et al., 2007; Baufreton et al., 2009)
*N* ^STN, GPe^	3	(Hahn and McIntyre, 2010)
	30	(Lindahl et al., 2013)
*G* ^CTX, STN^	0.125μ_scale_ nS	Result of optimization (1)
*G* ^GPe, STN^	1.11μ_scale_ nS	Result of optimization (2)
*G* ^STN, GPe^	15.8μ_scale_ nS	Result of optimization (3) for *N*^STN, GPe^ = 3
	1.5μ_scale_ nS	Result of optimization (3) for *N*^STN, GPe^ = 30
*G* ^MSN, GPe^	5.81μ_scale_ nS	Result of optimization (4) for *N*^STN, GPe^ = 3
	12.0μ_scale_ nS	Result of optimization (4) for *N*^STN, GPe^ = 30
*G* ^GPe, GPe^	0.44μ_scale_ nS	Result of optimization (4) for *N*^STN, GPe^ = 3
	0.21μ_scale_ nS	Result of optimization (4) for *N*^STN, GPe^ = 30

We introduced a scale factor of μ_scale_ = 0.85.

### 3.2. Glutamatergic and GABAergic inputs shape cortically evoked responses

Experimental studies explored the origin of the characteristic pattern of excitations and inhibitions in the PSTHs. In monkeys, incoming connections were blocked by local injection of GABA and glutamate antagonists (Kita et al., 2004; Polyakova et al., 2020). In our computational model, we created similar scenarios by cutting incoming connections to individual STN or GPe neurons. The resulting PSTHs are shown in Figure 4.

Cutting cortical inputs to single STN neurons led to a reduction of the amplitude of the early excitation in the response of these neurons to cortical stimuli (Figure 4A). Furthermore, their mean firing rate decreased. In responding GPe neurons, cutting cortical input to STN neurons reduced the amplitude of the early excitation substantially (Figure 4B).

Cutting all GPe inputs to the responding STN neurons strongly diminished the amplitude of the second excitation and the late inhibition. Furthermore, it increased the mean firing rate of STN neurons (Figure 4C). It also led to slow, damped oscillations of the instantaneous firing rate following the initial early excitation (Figure 4C). In responding GPe neurons, we also found an increase in the mean firing rate. Furthermore, the amplitude of the early excitation increased, and slow oscillations occurred after the second excitation (Figure 4D).

Finally, cutting striatal inputs to responding GPe neurons led to a reduction of the amplitude of the second excitation of responding STN neurons and to a shortening of the late inhibition (Figure 4E). In responding GPe neurons, it strongly suppressed the inhibition between early and late excitations (Figure 4F).

### 3.3. Spatio-temporal characteristics of cortical stimulation-evoked responses

Next, we studied the spatio-temporal characteristics of cortically evoked responses in the computational model. To this end, we analyzed the trial-averaged responses of BG neurons with different coordinates (see schematics in Figure 1).

In Figure 5, we show the trial-averaged instantaneous firing rate, *p*(*t, s*^X^), of a neuron with coordinate *s*^X^ in nucleus X. For comparison, we marked the mean firing rate of cortical neurons (4 Hz), STR MSNs (0.67 Hz), STN neurons (11.8 Hz), and of GPe neurons (30.4 Hz) on the color axes in Figure 5. The response of BG neurons strongly depended on their baseline firing rate and on |*s*^X^|, i.e., the coordinated difference to the stimulus center. Neurons with similar coordinates as the stimulated cortical neurons, *s*^X^≈0, possessed the characteristic responses presented in Figure 3. STN neurons with small |*s*^STN^| showed a pronounced late inhibition (Figure 5B). In the GPe, neurons with moderate |*s*^GPe^| showed a substantially shorter late excitation than GPe neurons with |*s*^GPe^|≈0. The dependence of cortically evoked responses on the coordinate *s*^X^ was more pronounced for large *N*^STN, GPe^ = 30 (Figures 4C, D).

Motivated by the impact of *N*^STN, GPe^ on the evoked responses of BG neurons, we studied the impact of the network structure on the distributions of single-neuron mean firing rates and the spatio-temporal characteristics of cortically evoked responses.

### 3.4. Network structure shapes distribution of single-neuron mean firing rates

Next, we analyzed how the network connectivity affected the dynamics of STN and GPe neurons. We considered three network structures: N-networks, D-networks, and S-networks. N-networks were obtained by implementing outgoing synaptic connections such that the presynaptic neurons connect to postsynaptic neurons with similar coordinates. D-networks were obtained in the same way, except that 10% of synaptic connections were randomly selected and displaced systematically (Model and methods). Lastly, S-networks were obtained like N-networks except that neurons were only allowed to project to every second neuron in the postsynaptic nucleus (see Model and methods for more details).

**Figure 6:**
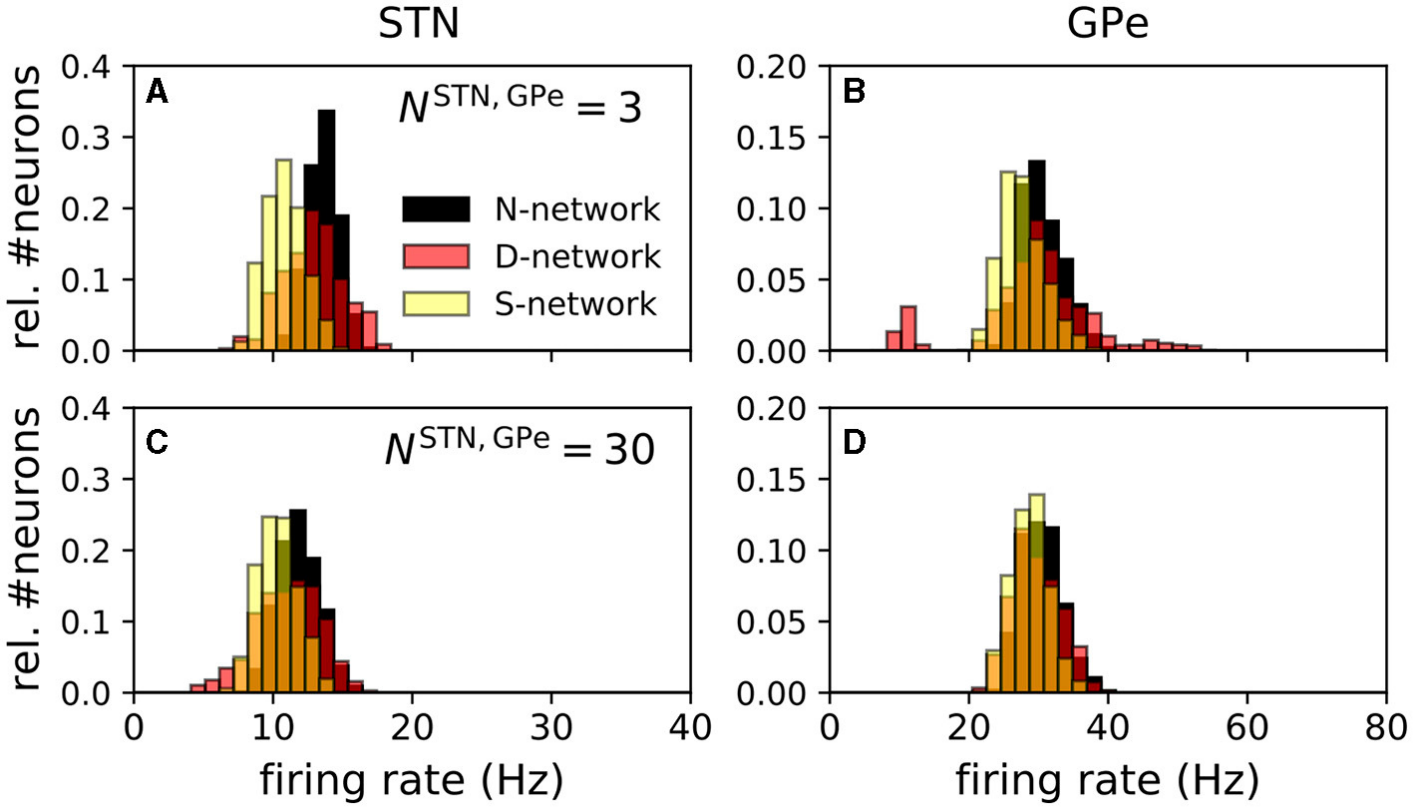
Distribution of single-neuron mean firing rates depended on network structure. Panels **(A–D)** show distributions of single-neuron mean firing rates for an N-network, a D-network, and an S-networks. The left column shows simulation results for STN and the right column results for GPe neurons. Panels **(A, B)** show results for *N*^STN, GPe^ = 3 and panels **(C, D)** for *N*^STN, GPe^ = 30. Estimates of single-neuron mean firing rates were obtained by counting the number of spikes in a simulated time window of 9 s. Prior to that a 31 s time window was simulated to ensure stationary dynamics. Mean firing rates of the inner 30 STN neurons ($-1/6<s_{i}^{\text{STN}}<1/6$) **(A, C)** and the inner 100 GPe neurons ($-1/6<s_{i}^{\text{GPe}}<1/6$) **(B, D)** for a total of 96 realizations of the noise and the Poisson input are shown.

Estimated distributions of single-neuron mean firing rates of STN and GPe neurons obtained from simulations of the computational model are shown in Figure 6. Firing rate distributions were unimodal except for D-networks and small *N*^STN, GPe^. For the latter, individual STN to GPe connections were strong, and random displacement of connections in the D-network led to variability in the number of incoming STN connections per GPe neuron. Few incoming connections resulted in low mean firing rates, whereas many incoming connections resulted in high mean firing rates. This led to the additional peaks in Figure 6B.

Following, we will restrict our analysis to networks with *N*^STN, GPe^ = 3, thereby modeling a high degree of specificity of STN → GPe connections as reported by experimental studies in monkeys (Shink et al., 1996). We continue by analyzing how the network structure affects the spatio-temporal pattern of evoked responses.

### 3.5. Network structure shapes evoked spatio-temporal responses

**Figure 7:**
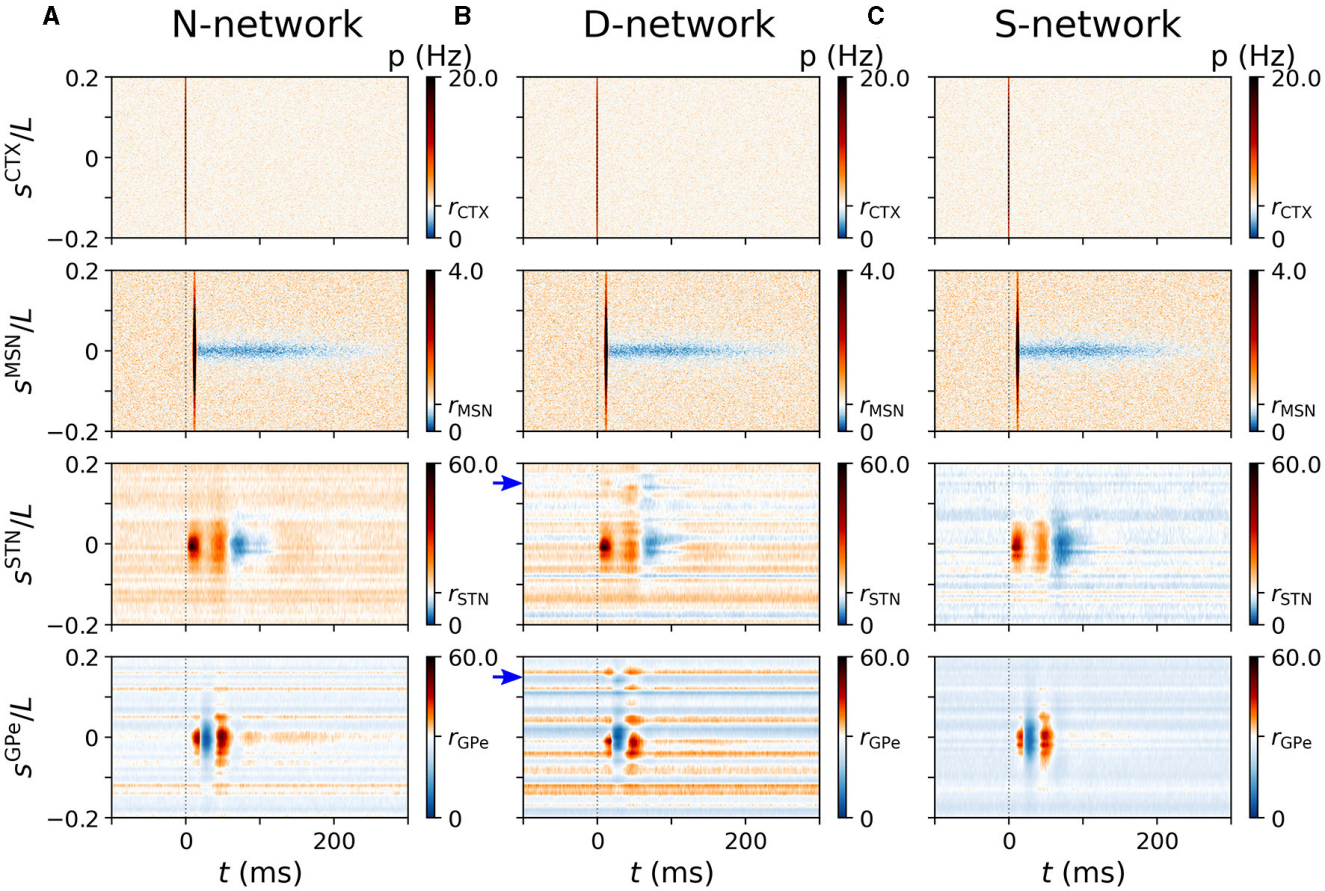
Representative cortically evoked spatio-temporal responses in an N-network, a D-network, and an S-network. Rows show spatio-temporal response patterns of CTX (top), MSN (second to top), STN (second to bottom), and GPe neurons (bottom). Columns **(A–C)** show results from simulations of the computational model for an N-network **(A)**, a D-network **(B)**, and an S-network **(C)**. Blue arrows mark displacement *d* = 0.15*L* in D-network. Results were averaged over 24 trials with different realizations of noise and Poisson input. For each trial results were averaged over a sequence of 500 stimuli. Cortical stimuli were centered at *s*^CTX^ = 0. Responses of individual STN and GPe neurons strongly depended on, |*s*^X^|, with X=STN,GPe (see also Figure 5).

We studied cortically evoked responses in N-networks, D-networks, and S-networks. Figure 7 shows simulated responses of BG neurons to cortical stimuli for the three network structures. Column A shows the results for the N-network from Figure 5B. In the D-network, a displacement of randomly selected connections by *d* = 0.15*L* led to additional responses of STN neurons near *s*^STN^ = 0.15*L* and responses of GPe neurons near *s*^GPe^ = 0.15*L* (Figure 7B). We also find that the evoked response of neurons with lower baseline activity deviated from the characteristic response patterns (blue horizontal lines in Figure 7B). In contrast, in an S-network, neurons showed less pronounced response patterns than in N-networks and overall reduced baseline activity (Figure 7C).

The strong variability of single-GPe neurons' mean firing rates in D-networks (see also Figure 6) can be seen in Figure 7B. While neurons at some coordinates, *s*^GPe^, fired at a low rate and responded only weakly to cortical stimuli, others were highly active and showed strong responses. In our computational model, this resulted in high trial-averaged instantaneous firing rates *p*(*t, s*^GPe^) for certain *s*^GPe^ and low *p*(*t, s*^GPe^) for others.

So far, our results suggest that alterations of the network connectivity lead to changes in the evoked spatio-temporal response pattern. We studied two types of alterations: first, in D-networks, we randomly selected 10% of the connections and exchanged the postsynaptic neurons by postsynaptic neurons at different locations (shifted by *d* relative to the original postsynaptic neuron). This led to a weaker response in the original target region and an additional response in another region. Second, in S-networks, the responding region was larger; however, responses to cortical stimulation were weaker as a whole.

### 3.6. BG responses to cortical two-site stimulation

Next, we delivered a sequence of pairs of priming and test stimuli to different cortical coordinates. This mimicked the stimulation of neuronal populations representing different features. In our computational model, this was implemented by delivering the priming stimulus to a cortical population centered at $s_{\text{I}}^{\text{CTX}}=-\text{Δ}s/2$ and the test stimulus to a population centered at $s_{\text{II}}^{\text{CTX}}=\text{Δ}s/2$ (Figure 2). The stimulus profiles were given by Equation (10).

**Figure 8:**
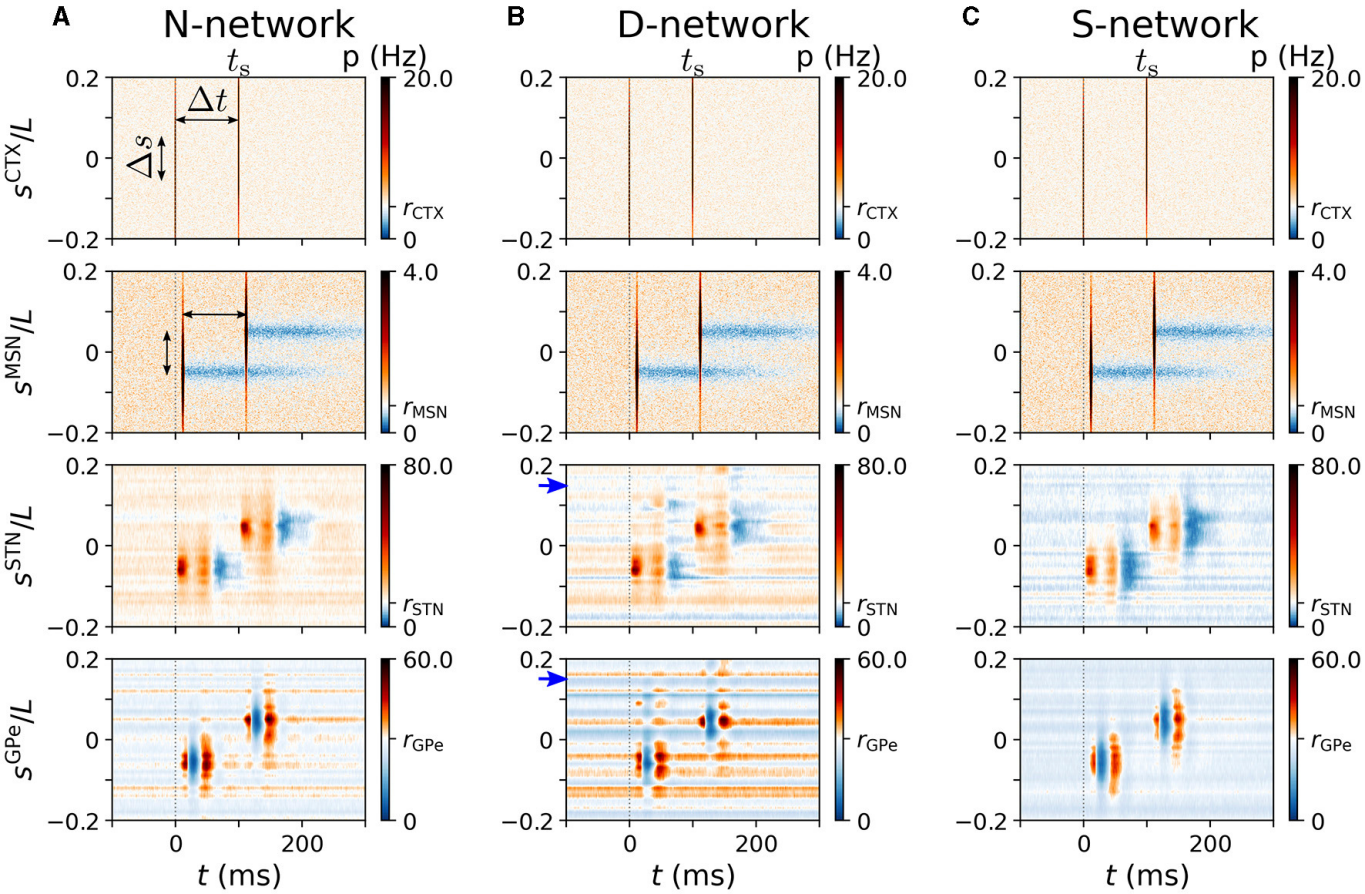
Spatio-temporal response to cortical two-site stimulation. Panels **(A–C)** show representative trial-averaged responses of simulated BG and cortical neurons at different locations, *s*^X^, to cortical two-site stimulation. Stimulation sites were at ±Δ*s*/2 and the inter-stimulus interval was Δ*t*. Column **(A)** shows results for an N-network, column **(B)** results for a D-network, and column **(C)** results for an S-network. Blue arrows mark displacement *d* = 0.15*L* in D-network. Rows show the instantaneous firing rates of neurons at location *s*^X^ in the cortex, the striatum, the STN, and the GPe (from top to bottom). Parameters: σ_s_ = 0.05*L*/π, Δ*s* = 0.1*L*, Δ*t* = 100 ms, *N*^STN, GPe^ = 3. Results were averaged over 500 stimuli and 24 realizations of noise and Poisson input.

Representative spatio-temporal responses for Δ*s* = 0.1*L* are shown in Figure 8. For a rather large time lag of Δ*t* = 100 ms, each stimulus caused spatio-temporal responses that were similar to the ones caused by a single stimulus in the respective network (compare Figures 7, 8).

Next, we performed a more detailed analysis of the response patterns. To this end, we compared two cases: (i) only the test stimulus was delivered to $s_{\text{II}}^{\text{CTX}}=\text{Δ}s/2$ at time *t*_s_ and (ii) the priming stimulus and the test stimulus were delivered: the priming stimulus to $s_{\text{I}}^{\text{CTX}}=-\text{Δ}s/2$ at time *t*_s_−Δ*t* and the test stimulus to $s_{\text{II}}^{\text{CTX}}=\text{Δ}s/2$ at time *t*_s_. Note that we only considered positive inter-stimulus intervals, Δ*t*>0. In what follows, we mark quantities corresponding to case (i) by the index “i” and quantities corresponding to case (ii) by the index “ii”.

For our analysis, we averaged the trial-averaged PSTHs of all neurons with coordinates that were close to $s_{\text{II}}^{\text{CTX}}$, i.e., *s*^X^∈[Δ*s*/2−*A*/2, Δ*s*/2+*A*/2], with X=STN,GPe. Here, *A* is the width of the coordinate range over which responses were averaged. In the case (i), only the test stimulus was delivered at time *t*_s_ to the stimulation site at Δ*s*/2. We denote the average response of BG neurons in nucleus X with *s*^X^∈[Δ*s*/2−*A*/2, Δ*s*/2+*A*/2] at time *t* as $F_{\text{(i)}}^{\text{X}}\left(t|\text{Δ}s/2\right)$. In case (ii), an additional priming stimulus was delivered to the stimulation site at −Δ*s*/2 at time *t*_s_−Δ*t*. We denote the average response of BG neurons in nucleus X with *s*^X^∈[Δ*s*/2−*A*/2, Δ*s*/2+*A*/2] at time *t* as $F_{\text{(ii)}}^{\text{X}}\left(t|\text{Δ}s/2,-\text{Δ}s/2,\text{Δ}t\right)$. We used *A* = 0.045*L*.

To study how much the presence of the priming stimulus alters the response evoked by the test stimulus, we evaluated two quantities. The first quantity was


(13) $$\begin{array}{l} L_{\text{base}}^{\text{X}}(\text{Δ}t,\text{Δ}s):=\int_{t_{\text{s}}-T^{-}}^{t_{\text{s}}}dt\;|F_{\text{(ii)}}^{\text{X}}\left(t|\frac{\text{Δ}s}{2},-\frac{\text{Δ}s}{2},\text{Δ}t\right)\\ \;-{F_{\text{(i)}}^{\text{X}}\left(t|\frac{\text{Δ}s}{2}\right)|}^{2}. \end{array}$$

Here, *T*^−^>0 is the time range prior to the test stimulus during which the change of the neurons' trial-averaged instantaneous firing rate due to the presence of the priming stimulus was evaluated. $L_{\text{base}}^{\text{X}}\left(\text{Δ}t,\text{Δ}s\right)$ measures how much the presence of the priming stimulus affects spiking of neurons in nucleus X shortly before their evoked response to the test stimulus. It therefore provides information on how much the baseline activity of neurons in nucleus X is affected by the priming stimulus. The second quantity we evaluated was


(14) $$\begin{array}{l} L_{\text{re}}^{\text{X}}(\text{Δ}t,\text{Δ}s):=\int_{t_{\text{s}}}^{t_{\text{s}}+T^{+}}dt\;|L_{\text{(ii)}}^{\text{X}}\left(t|\frac{\text{Δ}s}{2},-\frac{\text{Δ}s}{2},-\text{Δ}t\right)\\ \;-{L_{\text{(i)}}^{\text{X}}\left(t|\frac{\text{Δ}s}{2}\right)|}^{2}. \end{array}$$

$L_{\text{re}}^{\text{X}}\left(\text{Δ}t,\text{Δ}s\right)$ measures how much the presence of the priming stimulus affected the responses of neurons in nucleus X evoked by the test stimulus.

**Figure 9:**
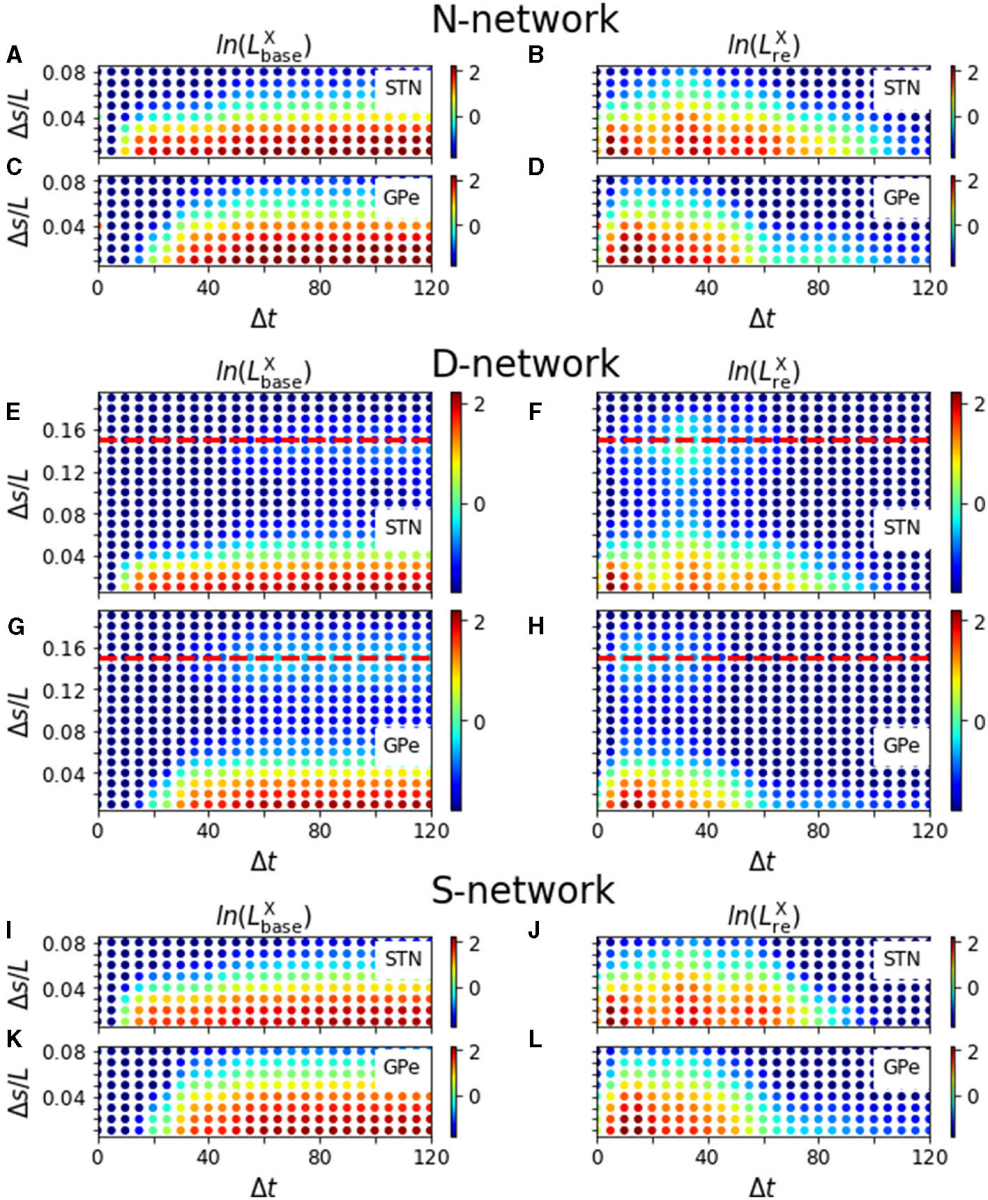
Modulation of evoked responses by a priming stimulus. Panels **(A–D)** show results for N-networks, panels **(E–H)** for D-networks, and panels **(I–L)** for S-networks. In the left column, we show the natural logarithm of $L_{\text{base}}^{\text{X}}\left(\text{Δ}t,\text{Δ}s\right)$ and in the right column the natural logarithm of $L_{\text{re}}^{\text{X}}\left(\text{Δ}t,\text{Δ}s\right)$ for *X* = STN and *X* = GPe, respectively. Parameters: *N*^STN, GPe^ = 3. PSTHs used for the calculation of $L_{\text{base}}^{\text{X}}\left(\text{Δ}t,\text{Δ}s\right)$ and $L_{\text{re}}^{\text{X}}\left(\text{Δ}t,\text{Δ}s\right)$ were averaged over sequences of 500 stimuli and over 24 realizations of noise and Poisson input. For D-networks, we used *d* = 0.15*L* (red, dashed line). *T*^+^ = 200 ms and *T*^−^ = 190 ms.

In Figure 9, we show results for $L_{\text{base}}^{\text{X}}\left(\text{Δ}t,\text{Δ}s\right)$ and $L_{\text{re}}^{\text{X}}\left(\text{Δ}t,\text{Δ}s\right)$ for an N-network, a D-network, and an S-network obtained from simulations of our computational model. $L_{\text{base}}^{\text{X}}\left(\text{Δ}t,\text{Δ}s\right)$ and $L_{\text{re}}^{\text{X}}\left(\text{Δ}t,\text{Δ}s\right)$ showed different dependencies on Δ*t* and Δ*s*. For short inter-stimulus intervals, Δ*t*, $L_{\text{base}}^{\text{X}}\left(\text{Δ}t,\text{Δ}s\right)$ was close to zero, indicating that the baseline activity prior to the test stimulus was not affected by the presence of the priming stimulus. For long inter-stimulus intervals, $L_{\text{base}}^{\text{X}}\left(\text{Δ}t,\text{Δ}s\right)$ increased and finally saturated for fixed Δ*s* as more and more of the response evoked by the priming stimulus impacts the baseline activity of neurons before their response to the test stimulus. The saturation for large Δ*t* indicates that the impact of the response evoked by the priming stimulus was over before neurons responded to the test stimulus. Additionally, increasing the coordinate difference, Δ*s*, between stimulated cortical subpopulations reduced the impact the priming stimulus had on neurons responding to the test stimulus in N-networks and S-networks. Accordingly, a characteristic width of functional channels in which cortical inputs are processed independently may be derived. In contrast, in D-networks $L_{\text{base}}^{\text{X}}\left(\text{Δ}t,\text{Δ}s\right)$ attained another local maximum as a function of Δ*s* when Δ*s* was close to the displacement, *d*, of synaptic connections (Figures 9E, G).

Remarkably, $L_{\text{re}}^{\text{X}}\left(\text{Δ}t,\text{Δ}s\right)$ showed a more complex dependence on Δ*t* and Δ*s* than $L_{\text{base}}^{\text{X}}\left(\text{Δ}t,\text{Δ}s\right)$. Several local maxima occurred at small Δ*s* and at Δ*t*≈5 − 10 ms, Δ*t*≈30 − 40 ms, and Δ*t*≈50 − 60 ms for STN neurons (Figure 9B) and at Δ*t*≈10 − 15 ms for GPe neurons. A comparison of these times with the PSTHs in Figure 3 suggested that they correspond to the timings of the two excitations and the gap in between in the PSTHs of STN neurons (Figure 3A) and the timing of the first excitation in the PSTHs of GPe neurons (Figure 3B). However, the delay between STN and GPe neurons needs to be considered (1 ms in simulations; however, it took about 5 ms for the postsynaptic neurons to respond to incoming excitatory input due to the finite time constant of the membrane potential).

Most remarkable, for all considered network structures, $L_{\text{re}}^{\text{STN}}\left(\text{Δ}t,\text{Δ}s\right)$ was more sensitive than $L_{\text{base}}^{\text{X}}\left(\text{Δ}t,\text{Δ}s\right)$ or $L_{\text{re}}^{\text{GPe}}\left(\text{Δ}t,\text{Δ}s\right)$ in the sense that the influence of the priming stimulus was measurable for larger Δ*s*, i.e., when stimulation sites were further apart. In particular, an inter-stimulus interval of about 30 − 40 ms between the stimulus deliveries led to the largest coordinate difference between stimulation sites for which the influence of the second stimulus was measurable using $L_{\text{re}}^{\text{STN}}\left(\text{Δ}t,\text{Δ}s\right)$ (Figures 9B, F, J). Note that this time interval also corresponded to the inter-stimulus intervals for which $L_{\text{re}}^{\text{STN}}\left(\text{Δ}t,\text{Δ}s\right)$ showed a local maximum for at Δ*s*≈*d* in D-networks (Figure 9F).

## 4. Discussion

Cortically evoked responses of STN and GPe neurons exhibit characteristic sequences of excitations and inhibitions that were observed in rodents (Kita and Kita, 2011) and primates (Nambu et al., 2000; Kita, 2007; Jaeger and Kita, 2011; Polyakova et al., 2020). We developed a computational model of the STN-GPe circuit that reproduced these response characteristics and related them to aspects of the topology of synaptic connections. Furthermore, we presented a one- and a two-site stimulation technique to quantify the width of functional channels in the BG network. Our results suggest that details of the synaptic connectivity are critical for the processing of cortical signals. They further support the use of computational models that include synaptic connectivity that is derived from experimental findings rather than random connections. The presented one- and two-site stimulation protocols enable probing of connectivity patterns in preclinical experiments. Based on our computational results on the effect of alterations of network connectivity on cortically-evoked responses, one may design preclinical experiments to falsify or verify our predictions. With such a combined computational and experimental approach, one may reveal further characteristics of synaptic connectivity in the BG and may identify patterns of synaptic reorganization in neurological diseases, e.g., Parkinson's disease.

The characteristic pattern of excitations and inhibitions in cortically evoked responses of STN and GPe neurons was studied experimentally (Nambu et al., 2000; Kita et al., 2004; Kita, 2007; Kita and Kita, 2011; Polyakova et al., 2020). Cortical stimulation triggered characteristic responses of STN neurons that consisted of an early excitation and a late excitation, which were separated by a gap, and a long, late inhibition (Nambu et al., 2000; Polyakova et al., 2020). In GPe neurons, responses showed an early excitation that was followed by an inhibition and a late excitation (Nambu et al., 2000; Kita, 2007; Jaeger and Kita, 2011). These features were well reproduced by our computational model (Figure 3).

Polyakova et al. (2020) found that local injection of glutamate receptor antagonists into the STN diminished the early excitation, and that the injection of muscimol (a GABA receptor agonist) into the striatum or the GPe diminished the late excitation. Their results supported the suggestions of earlier studies that the early excitation in the evoked response of STN neurons is caused by glutamatergic input via the cortico-STN hyperdirect pathway and the late excitation results from disinhibition due to GABAergic input via the cortico-striato-GPe-STN indirect pathway (Nambu et al., 2000). In our computational model, we modeled these experiments by cutting glutamate inputs to single STN neurons (to mimic the local injection of glutamate receptor antagonists) and by cutting inhibitory inputs to STN neurons (to mimic the local injection of GABA antagonists) (Figure 4). In accordance with Polyakova et al. (2020) we observed a suppression of the early excitation in the former case (Figure 4A) and a suppression of the late excitation in the latter case (Figure 4C). However, in our computational model, the latter case also led to damped beta oscillations in the instantaneous firing rate of STN neurons (Figure 4C). Such damped oscillations were not reported by Polyakova et al. (2020). However, Polyakova et al. reported high variability in the amplitude of the early and late excitations in the responses of STN neurons after local injection of a GABA antagonist. In our computational model, cutting GPe inputs to STN neurons destabilized the characteristic response pattern of excitations and inhibitions in both responding STN and GPe neurons (Figures 4C, D). A similar destabilizing effect may occur in the experiments and may cause high variability of the amplitudes of excitations among responding STN neurons (Polyakova et al., 2020). Together, these findings suggest that the GPe → STN connections are critical for the GPe-STN network to process cortical input and stabilize baseline activity. The results from our computational model further suggest that cutting STR MSN inputs to single GPe neurons would diminish the inhibition in the GPe neurons' responses (Figure 4F) and also reduce the amplitude of the late excitation in the evoked response of STN neurons (Figure 4E). These results are in accordance with other experiments by Polyakova et al. (2020) in which the injection of muscimol into the putamen reduced the amplitude of the second excitation in STN neurons substantially. Our results on the effect of cutting STR MSN inputs to GPe neurons are also in line with the experimental results of Kita et al. (2004); however, Kita et al. injected a GABA antagonist locally into the GPe, which also reduced GABAergic input from other GPe neurons and not only STR MSN input. In our computational model, only STR MSN inputs were cut, which resulted in a substantial weakening of the inhibition in the response of GPe neurons (Figure 4F). Furthermore, our computational results suggest that the late excitation in the GPe may be partly due to excitatory input from the STN and partly due to disinhibition after striatal inhibition. This is in accordance with previous results by Kita and colleagues (Kita et al., 2004; Kita, 2007).

The variability of the STN and GPe neurons' baseline firing rates contributed to the variability of single-neuron responses to cortical stimuli in our computational model. In more detail, the characteristic features of neuronal responses were most pronounced among neurons with high baseline activity (see Figures 5, 7). Unfortunately, it is difficult to compare the responses of STN or GPe neurons with low mean firing rates to cortical stimuli to experimental data, because such neurons were often excluded from the analysis in experimental studies (Kita et al., 2004; Kita and Kita, 2011; Polyakova et al., 2020).

N-networks and S-networks resulted in unimodal distributions of single-neuron mean firing rates for STN and GPe neurons (Figure 6). In contrast, in D-networks, baseline firing rates of GPe neurons showed a multimodal distribution (Figure 6B). The shapes of these distributions obtained from our computational model reproduced experimental data for STN neurons as observed in studies in rat brain slices qualitatively; a histogram of single-neuron firing rates of tonically spiking STN neurons in rat brain slices can be found in Beurrier et al. (1999). However, the mean firing rate in that study was higher than in our model, as our model was fitted to experimental data from anesthetized rats presented in Kita and Kita (2011). A histogram of single-neuron mean firing rates of GPe neurons can be found in Figure 8B of Miguelez et al. (2012). There, a significant portion of GPe neurons did not spike (≈25% in Miguelez et al., 2012), and the broad distribution of single-neuron mean firing rates of GPe neurons suggests high variability of single-neuron mean firing rates. In our model, GPe neurons with a small number of incoming STN connections possessed very low firing rates (Figure 6B). High variability of single-neuron mean firing rates occurred due to variability in the number of incoming STN → GPe connections, which was only realized in D-networks for a small number of STN → GPe connections, *N*^STN, GPe^ = 3. For the latter, individual connections were substantially stronger than for *N*^STN, GPe^ = 30 because parameters were adjusted to fit the STN and GPe firing rates to experimental data (Figure 5B). The random displacement of connections in D-networks led to broader distributions of single-neuron mean firing rates in D-networks than in N-networks or in S-networks.

Our results suggest that cortical stimulation results in complex spatio-temporal response patterns in the STN and GPe. These patterns result from the propagation of signals along the cortico-STN hyperdirect pathway and along the cortico-striato-GPe-STN indirect pathway (Figure 1B), and the convergence of these pathways onto the same regions in the STN and the GPe. In our computational model, these patterns strongly depended on the underlying structure of synaptic connections (Figure 7). Evidence from animal models suggests that the synaptic network structure in the BG nuclei is impaired in the dopamine-depleted state in animal models for Parkinson's disease (Fan et al., 2012; Miguelez et al., 2012; Chu et al., 2015; Pamukcu et al., 2020). Modulation of evoked responses of individual BG neurons in the dopamine-depleted state has been reported and analyzed by Kita and Kita (2011) in a rodent model for Parkinson's disease. The authors observed that the characteristic patterns of excitations and inhibitions were strongly affected by dopamine depletion. Our results suggest that dopamine depletion may also affect the spatio-temporal pattern of evoked responses and affect the structure of parallel functional channels in the BG.

In the present paper, we studied three types of networks: a base case with an intact functional channel structure and two cases in which this structure was perturbed. In the base case, neurons projected to neurons expressing similar features. This was realized in N-networks by connecting neurons with similar (spatial) coordinates *s*^X^ (Figure 1C1). This mimicked an intact somatotopic organization of synaptic connections (Nambu, 2011) and precise reciprocal loops between STN and GPe (Kita, 2007). In addition, we considered two types of altered network structures: First, in D-networks a shift in synaptic connectivity was induced for a fraction of the neurons such that they would connect to neurons with shifted (spatial) coordinates *s*^X^+*d* (Figure 1C2). Second, in S-networks, neurons projected to an enlarged area in the postsynaptic nucleus such that neurons with less similar coordinates were also targeted (Figure 1C3). The described changes in the network structure had a strong impact on cortically evoked spatio-temporal responses of STN and GPe neurons. While cortical stimulation in N-networks only triggered responses of neurons with similar coordinates, a second neuronal population expressing different features responded in D-networks. Interpreting these results, stimulation or activation of a cortical region corresponding to a certain body part or motor program would also activate neuronal populations representing different body parts or motor programs in D-networks. Such a perturbation of the BG structure may lead to the inability to activate these body parts independently. In contrast, in S-networks, cortical stimuli triggered less pronounced responses but of a bigger neuronal population. Such an alteration of the BG structure may correspond to less coordinated motor movements in response to cortical activation. Evidence from animal models suggests that a reorganization of network connectivity emerges in several BG related movement disorders, e.g., Parkinson's disease (Bronfeld and Bar-Gad, 2011). Experimental studies in the MPTP monkey model suggest that both types of impairment, i.e., responses to different body parts (D-network) and broadening of the projection region (S-network), may occur in the BG in Parkinson's disease. Boraud et al. (2000) reported that, under normal conditions, arm- and leg-related GPi neurons occurred in clusters and were linked to a single joint. In contrast, in the MPTP monkey model for Parkinson's disease, the overall number of responding neurons increased, and most responding neurons were linked to multiple joints (Boraud et al., 2000). Filion et al. (1988) reported that in MPTP monkeys, more globus pallidus neurons responded to the movement of a certain body part. Also, neuronal responses were elicited by the movement of more than one joint and by movements in different directions. Furthermore, some neurons responded to the movement of both upper and lower limbs on both the ipsi- and the contralateral sides. In contrast, in healthy animals, responses were only caused by the movement of a single joint on the contralateral side and in one direction (Filion et al., 1988). In rats, Cho et al. (2002) analyzed the reorganization in the lateral striatum (sensorimotor striatum) following 6-OHDA lesion. In controls, STR neurons that responded to the same body part were organized in clusters. However, after 6-OHDA lesion, the cluster size was reduced, and the portion of STR neurons that responded to more than one body part increased by a factor of 16 (Cho et al., 2002). These findings supported a hypothesis advanced by Mink (1996) that the BG's primary role may be the focused selection of the “correct” motor program and inhibition of competing ones. Synaptic reorganization in disorders such as Parkinson's disease would diminish action selection and cause motor symptoms.

Of particular interest for further analysis of the synaptic network structure of the BG would be to measure aspects of the synaptic connectivity in experiments. In earlier studies, the organization of cortico-STN connections was analyzed in tracer studies in monkeys (Monakow et al., 1978; Nambu et al., 1996). Furthermore, in Jeon et al. (2022), neuroanatomical techniques were used to construct 3D connectivity maps in mice and compare them to results from 7T MRI and tractography studies in humans. In the present study, we suggested a two-site stimulation protocol in which a test and a priming stimulus are delivered to two cortical stimulation sites at a certain distance and with a certain inter-stimulus interval. Analyzing how the priming stimulus influences the response of neurons to the test stimulus, we found that if the priming stimulus is applied long (>100 ms) before the test stimulus, varying the spatial distance between cortical stimulation sites yields an approximate “channel width,” characterizing the width of the cortical area in which stimulation activates the considered area in the STN or GPe (Figures 9A, C). This method may be used to analyze the spatial characteristics of the somatotopic organization in the BG. To realize two-site stimulation in an experiment, the response of single BG neurons to cortical stimuli would be measured, similar to the experiments performed by Nambu et al. (2000); Kita et al. (2004); Jaeger and Kita (2011); Kita and Kita (2011); and Polyakova et al. (2020). Then a priming stimulus would be administered with a time lag Δ*t* and at a distance Δ*s* from the original, test stimulus. Measuring the BG neuron's PSTH for a long sequence of stimuli and comparing it to the one in the absence of the priming stimulus yields estimates of the quantities $L_{\text{base}}^{\text{x}}\left(\text{Δ}t,\text{Δ}s\right)$ and $L_{\text{re}}^{\text{x}}\left(\text{Δ}t,\text{Δ}s\right)$. Evaluating, these quantities for different Δ*t* and Δ*s* yields similar data as the one shown in Figure 9 for each responding BG neuron.

The presented two-site stimulation protocol also allowed us to measure the displacement of synaptic connections in D-networks. In these networks, neurons in the considered region of the STN or GPe responded to stimulation of two distinct cortical regions (Figures 9E–H). Our computational results suggest that this method is most sensitive when the modulation of the evoked response of STN neurons by an earlier cortical stimulus rather than the modulation of their baseline activity is considered (Figures 9B, F, J), in particular for an inter-stimulus interval of about 30 − 40 ms. This time interval may be affected by synaptic transmission delays and needs to be verified experimentally. In our computational model, an approximate of the width of functional channels was also obtained if the modulation of the baseline activity of STN or GPe neurons by the presence of the cortical priming stimulus was studied (Figures 9A, E, I). This approach may also be realized by applying only one cortical stimulus and studying variations of neuronal activity from their baseline activity. However, the baseline activity may vary over time, whereas evoked responses possess characteristic features. In general, two-site stimulation may help to get a deeper understanding of the topology of synaptic connections in the BG, the somatotopic organization of the cortico-BG circuits, and to which extent this structure is impaired in animal models of neurological disorders, e.g., Parkinson's disease.

More detailed information on the organization of synaptic connections could inform future computational models. Current computational models often either consider random connectivity between nuclei, e.g., Lindahl et al. (2013); Ebert et al. (2014); Madadi Asl et al. (2022a); Adam et al. (2022); Salehi et al. (2023), or they incorporate parallel channels either by considering macroscopic parallel circuits of randomly connected subpopulations, e.g., Leblois et al. (2006); Fountas and Shanahan (2017), or by constructing parallel channels on the scale of the individual neurons, e.g., Terman et al. (2002); Hahn and McIntyre (2010); Lourens et al. (2015). The latter is somewhat comparable to the synaptic connectivity used in the present study. Our results suggest that differences in the organization of synaptic connections in computational models strongly impact cortically evoked responses and likely affect other characteristics of neuronal activity, such as synchronization or the existence of pathological oscillations. An accurate implementation of synaptic connections may be critical for generating clinically relevant hypotheses, e.g., about the response to brain stimulation.

Recently, Schmidt et al. (2020) related the shape of deep brain stimulation-evoked potentials to the involved pathways. While no information on somatotopic maps was revealed, the authors suggested that the shape of deep brain stimulation-evoked potentials may serve as a biomarker for adaptive deep brain stimulation, or may guide parameter selection and electrodes placement for deep brain stimulation in Parkinson's disease (Schmidt et al., 2020; Dale et al., 2022). Our results may motivate preclinical and clinical studies to use cortical as well as BG one- or two-site stimulation to analyze the spatial arrangement of synaptic connections between BG nuclei. Such insight may help to improve computational models of the BG and models on high-frequency deep brain stimulation as a treatment for medically refractory Parkinson's disease significantly.

To reduce complexity, we did not consider some aspects of the STN and GPe nuclei during the derivation of our computational model. For instance, recent experimental studies reported the existence of multiple neuron types with distinct functionality in the STN (Jeon et al., 2022) and the GPe (Mallet et al., 2012; Abdi et al., 2015; Mastro et al., 2017), which may affect network functionality such as the processing of cortical responses and rhythm generation (Suryanarayana et al., 2019; Gast et al., 2021). Neuron types in the GPe include prototypic neurons and arkypallidal neurons (Mallet et al., 2012; Abdi et al., 2015). While prototypic neurons have been found to project mainly to the STN and down-stream nuclei, arkypallidal neurons project to the striatum thereby providing feedback to this upstream nucleus. Anatomical studies also reported projections of STN neurons to the striatum (Beckstead, 1983; Kita and Kitai, 1987). Here, we neglected upstream synaptic connections of the STN-GPe circuit and focused on the most common neuron type in the GPe, i.e., prototypic neurons. There is also evidence from anatomical studies that STN neurons form local axon collaterals suggesting recurrent STN connections (Hammond and Yelnik, 1983; Gouty-Colomer et al., 2018); However, recent studies performing simultaneous multi-cell recordings in rat brain slices reported the absence of functional intra-STN connectivity (Steiner et al., 2019). Therefore, we did not consider synaptic connections between STN neurons in our computational model. Another simplification is the assumption of a one-dimensional arrangement of the neurons along the *s*^X^-axes. The STN and GPe are three-dimensional structures and evidence from experimental studies suggests non-homogeneous synaptic connectivity along different directions. For instance, STN axons form band-like terminal fields in the globus pallidus that are aligned with those of striatal axons (Hazrati and Parent, 1992). This would likely impact spatio-temporal characteristics of cortically evoked responses and the orientation of functional channels. Furthermore, the somatotopic organization of STN and GPe nuclei are more complex. For instance it includes multiple body maps for inputs from the primary motor cortex and the supplementary motor area, respectively (Nambu, 2011). Some experimental evidence also suggests that within regions that represent a certain body part neurons encoding similar motor features are sometimes spread out across a larger area instead of clustering together (DeLong et al., 1985). Further studies are required to understand how these factors impact the spatio-temporal response patterns evoked by cortical stimulation studied in the present paper.

As explained above, the functional channels used in this study are related to the impact, specifically spatial range and coverage of electrical stimuli on parts of brain circuits (see Figure 2). These functional channels are not meant to be building blocks of a neural code as, e.g., activity sequences corresponding to sub-second behavioral motifs (Markowitz et al., 2018). However, disease-related changes as reflected by the width of these functional channels may impact behaviorally relevant activity sequences. Accordingly, functional channels may help elucidate neuronal information processing under physiological as well as pathological conditions.

In a future study, we want to address how characteristic measures, such as the width of functional channels, can be harnessed to calibrate multisite deep brain stimulation, for instance, coordinated reset stimulation (Tass, 2003; Tass et al., 2012; Adamchic et al., 2014; Wang et al., 2016, 2022; Bore et al., 2022), random reset stimulation (Kromer and Tass, 2020; Khaledi-Nasab et al., 2021a), and other multisite stimulation protocols (Khaledi-Nasab et al., 2021b, 2022; Weerasinghe et al., 2021; Kromer and Tass, 2022) for improving desynchronizing effects, especially in the presence of reorganized somatotopic maps. In computational studies, the desynchronization effect of coordinated reset stimulation was more pronounced when individual stimuli were delivered to segregated neuronal subpopulations (Popovych and Tass, 2012; Lysyansky et al., 2013; Ebert et al., 2014; Zeitler and Tass, 2015) suggesting that effective stimulation requires appropriate spacing of stimulation sites, e.g., minimal distances between the latter. This is in accordances with results from preclinical studies on coordinated reset deep brain stimulation in the MPTP monkey model, where weaker stimulation led to more pronounced long-lasting effects (Tass et al., 2012), and findings obtained in a clinical study on acoustic coordinated reset stimulation in tinnitus patients, where larger gaps between stimulus frequencies were correlated with better acute reduction of tinnitus loudness and annoyance after 16 min of sound treatment (Tass et al., 2019; Munjal et al., 2021). Future computational and pre-clinical studies might use the functional channel width, as introduced here, to determine optimal stimulation site spacing.

## Data availability statement

The original contributions presented in the study are included in the article/supplementary material, further inquiries can be directed to the corresponding author.

## Author contributions

JK, HB, and PT conceived the idea and designed the study, analyzed and interpreted the numerical data, and revised and finalized the manuscript. JK performed the numerical simulations and prepared the initial draft. All authors contributed to the article and approved the submitted version.
